# The Tumor Microenvironment: A Milieu Hindering and Obstructing Antitumor Immune Responses

**DOI:** 10.3389/fimmu.2020.00940

**Published:** 2020-05-15

**Authors:** Alireza Labani-Motlagh, Mehrnoush Ashja-Mahdavi, Angelica Loskog

**Affiliations:** ^1^Department of Immunology, Genetics and Pathology, Uppsala University, Uppsala, Sweden; ^2^Department of Molecular Biology, Umeå University, Umeå, Sweden

**Keywords:** tumor, tumor microenvironment, immune cells, immune response, immunosuppression, antitumor

## Abstract

The success of cancer immunotherapy relies on the knowledge of the tumor microenvironment and the immune evasion mechanisms in which the tumor, stroma, and infiltrating immune cells function in a complex network. The potential barriers that profoundly challenge the overall clinical outcome of promising therapies need to be fully identified and counteracted. Although cancer immunotherapy has increasingly been applied, we are far from understanding how to utilize different strategies in the best way and how to combine therapeutic options to optimize clinical benefit. This review intends to give a contemporary and detailed overview of the different roles of immune cells, exosomes, and molecules acting in the tumor microenvironment and how they relate to immune activation and escape. Further, current and novel immunotherapeutic options will be discussed.

## Introduction

The tumor microenvironment (TME) has a decisive role in tumor differentiation, epigenetics, dissemination, and immune evasion. In fact, the TME is a highly heterogeneous milieu consisting of different cell types and many abundant molecules produced and released by tumor cells, stromal cells, and immune cells. In this review, the TME will be discussed from the immunologist's point of view.

Concrete evidences support that both innate and adaptive immunity are involved in immune surveillance. This is referred to as the elimination phase and is reviewed elsewhere (1). If the transformed cells evade immune control during the elimination phase, tumors are formed. As tumor cells and its stroma progress, the immunosuppressive mechanisms increase in magnitude. Although tumor cells can be recognized and eliminated by the immune system, the tumor continues to grow (equilibrium phase) and escapes surveillance (escape phase) later. Lymphocytes including natural killer (NK) cells, CD8^+^ T cells and CD4^+^ helper T (Th) cells together with proinflammatory macrophages (M1) and dendritic cells from the anti-tumor immune responses while the heterogeneous population of myeloid-derived suppressor cells (MDSCs) and Foxp3^+^ regulatory T cells (Tregs) counteract tumor immunity. These cells are attracted and expanded by the tumor and its stroma to both control the present effector lymphocytes and to hamper the novo activation of tumor-reactive lymphocytes. Tumor cells evade the immune cells by a plethora mechanisms such as downregulation or loss of tumor antigens, releasing immunosuppressive extracellular vesicles including exosomes, releasing immunosuppressive molecules including IL-10 and transforming growth factor β (TGF-β), shedding soluble major histocompatibility complex (MHC)-I, loss of adhesion molecules such as ICAMI, developing resistance to apoptosis by upregulation of BCL-2 and other anti-apoptosis molecules, and overexpressing programmed death ligand 1 (PD-L1) as well as Fas ligand and tumor necrosis factor (TNF)-related apoptosis-inducing ligand (TRAIL). Tumor-released molecules shape the TME and induce immunosuppression that debilitate robust antitumor immune responses.

**Figure 1:**
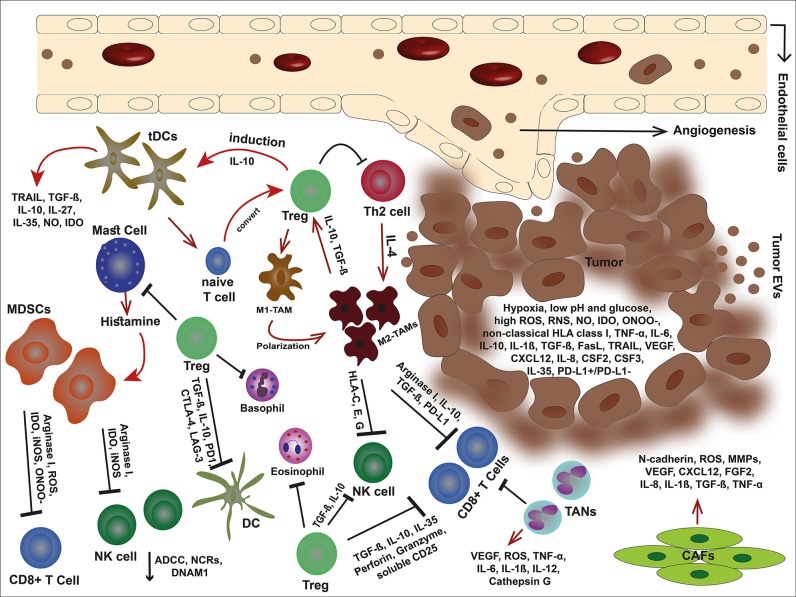
An overview of tumor microenvironment. In the immunosuppressive TME, malignant cells debilitate the antitumor immune responses through secretion of offensive and detrimental molecules, collaboration with cancer-associated stromal cells, and exploit immune scape mechanisms to outwit the immune cells. Tumor cells alter their milieu by lowering pH and glucose but high production of VEGF, non-classical HLA class I, death ligands such as FasL and TRAIL, anti-inflammatory cytokines, and metabolites such as IDO, ROS, RNS, ONOO^−^, and NO. These molecules can not only inhibit the immune cells but also elicit the stroma cells and facilitate tumor development. The cancer-associated stroma cells favor tumor cells by suppressing the immune responses and even induce each other. Tregs are capable to inhibit effector immune cells, eosinophils, basophils, and mast cells. Mast cells themselves induce MDSCs by releasing histamine. Tregs also stimulate tDC via IL-10 and impose M1-TAM polarization into M2-type. In turn, M2-TAMs eliminate effector cells via non-classical HLA class I, arginase I, IL-10, TGF-β, and PD-L1. In addition, MDSCs hinder effector cells by releasing arginase I, and metabolites such as IDO, ROS, ONOO^−^, and iNOS. TANs are other players that eliminate CD8+ T cells. The condition becomes more complicated with CAFs that promote angiogenesis, tumor growth, and invasion.

Dendritic cells (DCs) are key regulators of the immune system and orchestrate the immune reactions in the tumor but other innate immune cells play important roles as well. For example, M1 macrophages conduct tumor killing by releasing lytic enzymes, TNF-α, oxygen and nitrogen intermediates, as well as mediating antibody-dependent cell-mediated cytotoxicity (ADCC) (2–4). Basophils promote the infiltration of CD8^+^ T cells in the inflamed tumor tissue by secreting chemokines such as CCL3 and CCL4 (5). Eosinophils recruit in solid tumor due to the molecules released by tumor cells (6). Eosinophils not only regulate T-cell activation but also exert anti-tumor responses through degranulation and secretion of granzyme A as well as TNF-α via their natural killer group 2 member D (NKG2D) binding to NKG2D ligands (NKG2DLs) on tumor cells (7, 8). Finally, the N1 type of neutrophils can participate to activate anti-tumor T cell responses, induce tumor cell death by TRAIL, releasing reactive oxygen species (ROS), and participating in ADCC. Figure 1 shows an overview of the complexity in the TME.

Such a complex condition challenges effective therapies against the established cancer. While traditional therapies such as chemo- and radiotherapy target the tumor directly, novel options such as the wide range of immunotherapies, generally target the microenvironment and specifically the immune system. Hence, immunotherapeutics indirectly kill tumor cells via modulation of the TME and/or effector immune cells. Mapping the cell types and molecules present in the inimical tumor milieu will support the development of more effective treatments and teach us how to combine current available options.

## Dendritic Cells

Tumor immunity is formed in the same manner as immunity against virally-infected cells but with the lack of virus-mediated Toll-like receptor (TLR) activation. In brief, immature DCs in the TME effectively engulf and process tumor antigens. Damage-associated molecule patterns (DAMPs) elicit DC maturation and activation in which they reduce the ability to capture antigens and gain capacity to present them to lymphocytes. Mature DCs migrate to the lymph nodes to activate CD4^+^ and CD8^+^ T cells via antigen presentation on MHC class II and I molecules, respectively. They also provide co-stimulatory molecules such as B7 and TNF family members as well as cytokines for full T cell activation. Mature DCs can also increase the activation status of NK cells by their release of cytokines such as IL-12. Very recently, it was elucidated that infiltrating conventional DCs in the TME contribute to antitumor immune responses by selectively release of IFN-λ1 that stimulates Th1 differentiation and activation and effector CD8^+^ T-cell activation together with enhanced IFN-γ production through IL-12p70 production which as a result increases overall survival in human cancers (9).

Despite the capacity to mount anti-tumor immunity, the immune system fails to control the growing tumor. Tumor immune escape is as complex as immune activation. A crucial event is the lack of proper DC stimulation. If the DCs patrolling the TME are not properly matured, they will present tumor antigen in a tolerogenic fashion resulting in anergic/tolerant T cells. In the TME, regulatory or tolerogenic DCs (tDCs) display low expression of costimulatory molecules such as CD80 and CD86 as immature DCs, but they simultaneously have high expression of inhibitory molecules such as PD-L1 and cytotoxic T lymphocyte associated antigen-4 (CTLA-4). In fact, tDCs express various immunomodulatory factors (e.g., TRAIL, PD-L1, DC-SIGN, and galactin-1) and immunosuppressive molecules (e.g., TGF-β, IL-10, IL-27, NO, and IDO) (10). The tDCs can be induced by products of pathogens, VEGF, tumor-released cytokines (e.g., TGF-β and IL-10), and IL-10-secreting Tregs. Moreover, immature DCs that uptake apoptotic/necrotic DCs convert into tDCs with enhanced TGF-β secretion (11). Besides hampering activation of cytotoxic and T helper cells, IL-10-releasing tDCs elicit the differentiation of type 1 Tregs (Tr1) through immunoglobulin-like transcript 4 (ILT4)/HLA-G signaling pathway as well as inducing Foxp3^+^ Tregs from naïve CD4^+^ T cells (12).

## Tumor-Infiltrating Lymphocytes

Tumor-infiltrating lymphocytes (TILs) are certain lymphocytes infiltrating tumor site in response to molecular signals. The subsets include different T cell subsets including Tregs, innate lymphoid cells (ILCs) such as NK cells and NKT cells. The effector T cells and NK cells are frequently evoked and recruited to the TME for eliminating cancer cells through targeting tumor antigens and membrane ligands. The extravasation of TILs into the tissue is facilitated by selectins, integrins, and chemokines. Chemokines released by tumor cells and its stroma influence the immune cell infiltration, tumor cell proliferation, and metastasis (13). For example, CXCL16 and its receptor, CXCR6, enhance the infiltration of CD4 and CD8 T cells in colorectal cancer (14). CCL22 mediates Treg accumulation in the TME as shown in breast and ovarian cancer (15). Nevertheless, penetration of TILs into the tumor parenchyma considerably diminishes due to dysregulated vasculture that has down-regulated the receptors important for TIL attachment, rolling, and transmigration. Further, the chemokine profile can be reverted due to M2 macrophages and other immunosuppressive immune cells that together contribute to attract additional immunosuppressive cells while blocking TILs (16). TILs are present in the TME to defy the transformed cells. It is corroborated that a high level of CD3^+^, CD4^+^, and CD8^+^ TILs is associated with improved overall survival rates in cancer patients (17) while high levels of FoxP3^+^ TILs are negatively associated with overall survival (18). Both anti-tumor-reactive and tumor-supportive TILs are discussed below.

## CD8^+^ T cells

Naïve CD8^+^ cells are activated into cytotoxic T cells (CTLs) by mature DCs loaded with tumor antigens and trafficking to the tumor-draining lymph nodes as discussed previously. Infiltration into the TME is a result of several signaling molecules such as chemokines (e.g., CCL3, CCL4, CCL5, CCL20, CXCL9, CXCL10, CXCL11, and CXCL16) and their receptors (19). CCL5 released by the tumor and CXCL9 released by APCs in response to IFN-γ seems crucial since CCL5^hi^ CXCL9^hi^ tumors are highly infiltrated with TILs and respond to checkpoint blockade inhibition (CPI) therapy (20). CTLs target tumor cells via T cell receptor interaction with MHC class I and kill tumor cells through apoptosis induction. Apoptosis can be initiated via death receptor ligation, FasL and TRAIL, but also by secreting perforin and granzyme B. To avoid CTL-mediated killing, tumor cells down-regulate MHC class I but also undergo mutations that reduces the antigen processing and presenting capacity. Further, the tumor cells commonly upregulate anti-apoptotic molecules such as BCL-2 and/or have dysregulated expression of death receptors such as Fas. As a self-regulatory mechanism, CTLs upregulate checkpoint receptors including PD-1, CTLA-4, LAG-3, and TIM3 that makes them susceptible of inhibitory signaling since binding of checkpoint receptors induces CTL exhaustion and anergy (21).

## CD4^+^ T cells

Naïve CD4^+^ T cells encountering tumor antigens presented by APCs can differentiate into different cell subsets including Th1, Th2, Th9, Th17, Th22, and Tregs. Th1 cells secrete IL-2 and IFN-γ to provide help to CTL activation. However, IFN-γ also suppresses tumor growth, stimulate MHC upregulation and can suppress angiogenesis (22, 23). Th2 cells release cytokines such as IL-4 and IL-13 that can induce eosinophil recruitment into the TME which may participate in the antitumor response (24). However, CD4^+^ Th2-mediated responses may promote tumor growth via increasing angiogenesis and hampering Th1 cell-mediated immunity. Th17 cells exhibit dual roles in the TME. This cell subset elicit angiogenesis as well as tumor growth via pro-inflammatory IL-17A secretion and hamper antitumor responses by releasing immunosuppressive adenosine (25). Moreover, Th17 cells may convert into Treg cells due to the microenvironment to support immunosuppression in the tumor (25). Furthermore, abundant of Th17 cells in the tumor associate with poor prognosis (25). Nevertheless, it has been illustrated that the elevated number of Th17 cell population in ovarian tumors correlated to better survival rates (25). In fact, the association of chronic inflammation with tumor progression has been shown a long time ago (26). Both Th2 and Th17 contribute to chronic inflammation which promotes both tumor transformation and progression. Th22 cells secrete IL-22 that share similar structure with IL-10. The expression of this cytokine is not restricted to Th22 cells. Th17 subset and NK cells also produce IL-22 (27). Moreover, γδT cells co-express IL-22 and IL-17A (27). IL-22 promotes tumor cell proliferation through signal transducer and activator of transcription (STAT) 3 (27). STAT3 activation favors immunosuppressive activities in many immune cells such as DCs and macrophages. Hence, the enhanced number of tumor-infiltrating Th22 cells is associated with tumor progression. The nature of Th9 cells is still controversial. It was described that they are a distinct CD4^+^ T cell subset (28) while a contradictory study posed that Th9 cells are merely a subpopulation of IL-9^+^ Th2 cells (29). IL-9 is not restricted to Th9 cells but is expressed by subsets of Th2, Th17, ILC2 cells, Tregs, NKT cells, and mast cells (30). IL-9 can stimulate STAT1, 3, and 5 which are involved in many biological processes. Murine tumor-infiltrating CD4^+^ Th9 cells were shown to kill advanced tumors because of their ability for expressing cytolytic granzymes (28). In addition, Th9 cells significantly enhance CD8^+^ T cell trafficking into the TME *in vivo* (28).

## NK Cells

**Figure 2:**
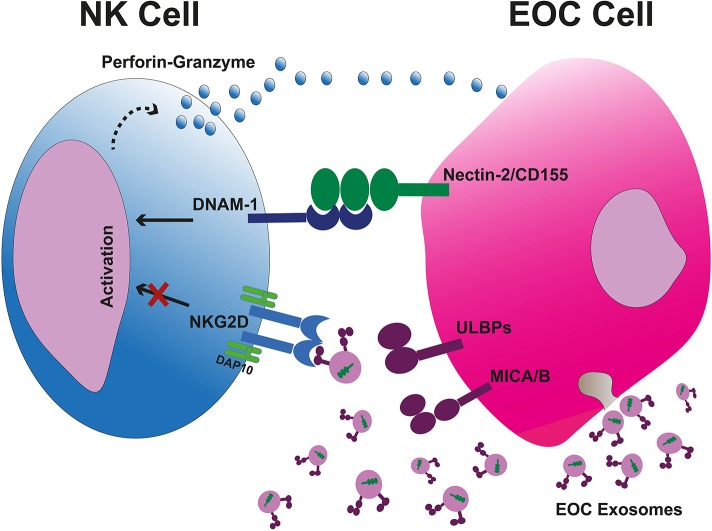
Tumor-cell escape and NK cell-mediated cytotoxicity. Tumor cell-released immunosuppressive exosomes expressing surface NKG2DLs impair the NK cell-mediated recognition and cytotoxicity. The exsosomes released by tumor cells internally carry the DNAM-1 ligands therefore they are not capable to bind the DNAM-1, leaving this activating receptor free to bind to its correlated ligands on tumor cells and kill them through apoptosis due to releasing perforin and granzyme B. EOC, epithelial ovarian cancer.

Like CTLs, NK cells kill tumor cells via induction of apoptosis which is primarily done by releasing cytolytic granules containing perforin and granzymes. NK cells are not MHC dependent. Instead, they have a range of activating and inhibiting receptors that regulate their killing capacity. Inhibiting receptors recognize for example MHC-I which restricts their killing of normal, healthy cells while activating receptors trigger cytolytic function. Activating receptors transduce signals through immunoreceptor tyrosine-based activation motif (ITAM) located in their cytoplasmic tail. These activating receptors include NKG2D, DNAX accessory molecule 1 (DNAM-1), NKp30, NKp44, and NKp46. NKG2D is also expressed on other cell types such as NKT cells, CD8^+^ αβT cells, and γδT cells (31). NKG2D ligands in human belong to two families; the MHC class I chain-related antigens A (MICA) and B (MICB) as well as the cytomegalovirus UL-16-binding proteins (ULBP) 1-6. These ligands are expressed on infected cells and on DNA damaged or transformed cells but in exiguous levels on different healthy cells (32). Upon NKG2D receptor-ligand binding, signal transduction culminates in degranulation of NK cells to eliminate tumor cells. NK cells are important in tumor control as a low activity of NK cells has been associated with increment of cancer risk (33). However, tumor cells downregulate their surface ligands to hamper the anti-tumor recognition to escape NK cell-mediated immune surveillance. The ligand downregulation is promoted by TGF-β, IFN-γ, STAT3, hypoxia, proteolytic shedding, and forming soluble ligands, as well as certain micro RNAs (i.e., miRNA-20a, miRNA-106b, miRNA-93, miRNA-373, and miRNA-520d) (34–38). Cancer cells also release immunosuppressive microvesicles including exosomes expressing surface NKG2DLs to obstacle the NKG2D receptors and block the tumor recognition (39). Nevertheless, NK cells exert DNAM-1 (CD226)-mediated tumor recognition if the tumor cell expresses DNAM-1 ligands to overcome the NKG2D blockade. DNAM-1-mediated killing is very effective since there are no soluble or vesicle-bound DNAM-1 ligands. The DNAM-1 ligands are internally packed into tumor-derived exosomes and are not exposed to NK cells (Figure 2) (39). Nevertheless, tumor-infiltrating NK cells (TINKs) are also affected by the TME and display: (1) altered polarization and phenotype, (2) increased expression of angiogenic factors such as VEGF, (3) reduced IFN-γ, (4) malfunction of degranulation and cytotoxic ability, (5) down-modulated CD16, NKG2D, and DNAM-1 (40, 41). It has been described that CD11b^high^ CD27^high^ NK cells can be converted into MDSCs in the TME due to GM-CSF (42). Although NK cells as cytotoxic innate lymphoid cells (ILCs) have a pivotal role in eliminating tumor cells, other subpopulations of ILCs show dual roles. These cells present mostly in the mucosae and mucosal-correlated lymphoid tissues. Non-cytotoxic ILCs fall into three groups comprising T-bet^+^ ILC1 (releasing TNF-α and IFN-γ), GATA3^+^ ILC2 (secreting IL-4, IL-5, IL-9, and IL-13), and RORγt^+^ ILC3 (CCR6^+^ cells releasing IL-17A, IL-22, GM-CSF, and CCR6^−^ cells secreting TNF-α, IFN-γ, IL-22, and GM-CSF) (43). Interestingly, ILC2 and ILC3 subsets may transdifferentiate into ILC1 cells and vice versa (44). Therefore, they can acquire or lose certain types of cytokines. It has been indicated that an enhanced number of RORγt^+^ ILC3 cells is associated with increased lymph node metastasis (45). In contrast, NKp46^+^ ILC3 cells indicated supportive antitumor response in a mouse melanoma (B16) model in an IL-12-mediated fashion (44). Nevertheless, TGF-β-releasing cancer cells convert NK cells into ILC1 cells in the TME as an immune escape mechanism (46).

## NKT Cells

After development from lymphoid precursors, NKT cells mature in the thymus. In human, NKT cells are few and form about 0.2% of peripheral blood T cells (47). The number of NKT cells becomes even lower in advanced cancer patients (47). Regardless of the low number, NKT cells may have an important function to eliminate tumor cells if they can infiltrate into tumor since the survival rate was higher in cancer patients with infiltrated NKT cells compared to those with no infiltration (48). The recruitment of NKT cells to the tumor might be due to the presence of lipid antigens that are essential for NKT-cell development; however, the role of such antigens in this regard is not clear (49). Nevertheless, it was shown that the recruitment of NKT cells can be mediated by CCR2, CXCR6, and leukocyte function associated antigen-1 (LFA-1) expressed on these cells (49). NKT cells not only show NK cell features such as expression of CD56, Fc receptor CD16, and granzyme production, but also display αβ-TCR to recognize both endogenous and exogenous lipid antigens presented on CD1d, a non-classical antigen-presenting molecule resembling MHC class I. CD1d is expressed on APCs, epidermal keratinocytes, hepatocytes, adipocytes, thymocytes, and is downregulated in tumor cells (50). Upon induction and activation through αβ-TCR and CD1d interaction, NKT cells release cytokines of both Th1 and Th2 type. Examples of the cytokines released by NKT cells include IFN-γ, TNF-α, IL-2, IL-4, IL-5, IL-6, IL-10, IL-13, IL-17 IL-21, TGF-β, and granulocyte monocyte-colony stimulating factor (GM-CSF) (51). Unlike type II subsets that have immunoregulatory function as for example seen in B-cell lymphoma mice to support MDSCs (52), type I NKT cells have antitumor activity through CD1d-dependent or—independent manner. The antitumor activity of type I NKT cells includes the regulation of effector cells via cytokines and tumor lysis via FasL, TRAIL, perforin, and granzymes (47). In addition, NKT cells expressing NKG2D can eliminate NKG2DL-expressing tumor cells. Nevertheless, interaction between type I NKT cells and Tregs showed the inhibition of NKT cells by Tregs via direct contact and providing help to Tregs by NKT cells via IL-12 secretion (53).

## Tumor-Associated Macrophages

Among tumor-associated immune stroma, tumor-associated macrophages (TAMs) have crucial functions and promote tumor progression. There is a correlation between TAM high densities with poor overall survival rate in patients with several cancer types except colorectal cancer (54, 55). Circulating monocytes migrate into the TME in response to different molecules such as growth factors [vascular endothelial growth factor (VEGF), colony-stimulating factor 1 (CSF-1), and granulocyte/macrophage CSF (GM-CSF)], cytokines (IL-6, IL-10 and TGF-β), and chemokines (e.g., CCL2, CCL3, CCL4, CCL7, and CCL8) (56–59). These molecules are released by different cell types including tumor cells in the TME. After residing, monocytes differentiate into TAMs depending on certain signaling molecules in the TME such as IL-6 and leukemia inhibitory factor (60). The induction of TAM differentiation is rendered by pro-inflammatory and anti-inflammatory cytokines, chemokines, growth factors, and hypoxia. TAMs are classified into two phenotypes, pro-inflammatory M1 type and anti-inflammatory M2 type, with tumor suppressive and tumor supportive function, respectively. The M2 macrophages undergo further polarization to become a few more subtypes including M2a, M2b, M2c, and M2d. This division is based on a variety of stimuli (61). Strikingly, M2 TAMs undergo metabolic reprogramming such as glycolysis activation, fatty acid synthesis, modification of tricarboxylic acid and nitrogen cycle metabolism (62). In addition to low expression of MHC class II, the mannose receptor and stabilin-1, tumor-educated M2 TAMs acquire immunosuppressive functions and promote tumor progression by releasing VEGF, MMP-7, MMP-9, IL-12, high levels of IL-10, TGF-β, hepatocyte growth factor and basic fibroblast growth factor (bFGF), adrenomedullin, urokinase-type plasminogen activator (uPA), thymidine phosphorylase (TP), prostaglandin E2 (PGE2), and semaphoring 4D (59, 63, 64). Unlike M1 phenotype, M2 TAMs participate in immune suppression, tumor migration, invasion and angiogenesis. Indeed, M2 TAMs induce angiogenesis by releasing VEGF, IL-1, TNF-α, TP, PGE2, adrenomedullin, and semaphoring 4D. They mediate the degradation of the ECM by uPA, MMP-7, and MMP-9. In the TME, M2 cells crosstalk to other cell types. They interact with tumor cells, MDSCs, Tregs, CAFs, Th2 cells, CD8^+^ T cells, and NK cells. In particular, tumor cells are involved in M2-like polarization by secreting lactic acid, a function interceded by hypoxia-inducible factor (HIF1α) (65). In turn, the tumor growth is influenced by TAM-releasing arginase 1 (Arg-1) induced by the lactic acid (65). Indeed, L-arginine depletion by arginase is culminated in the downregulation of the TCR ζ chain (66). TAMs induce the suppression of CD3-ζ chain expression in tumor-infiltrating T cells through oxidative stress (67). Thus, the TCR becomes non-functional due to loss of the CD3-ζ chain. TAMs also promote tumor progression through releasing molecules such as VEGF, IL-8 and IL-6 (68, 69). TAMs influence on tumor cell invasiveness when tumor-released TNF-α elicits the expression and secretion of CCL8 from TAMs (70). This chemokine not only upregulates the expression of CSF-1 from tumor cells but also recruits the circulating monocytes that express CCR2 (70). Furthermore, TAM secrete cytokines such as CCL22 and CCL20 to recruit CCR4^+^ and CCR6^+^ natural Treg (nTreg) cells, respectively (71). However, CCL22-expressing TAMs and tumor cells can be targeted and perished by CCL22-specific T cells (72). Macrophages expressing HLA-C, -E, and -G in both soluble and membrane forms can eliminate NK cells expressing NKG2 as well as the inhibitory leukocyte immunoglobulin-like receptor LIT-2 that consequently inhibit IFN-γ secretion (71). Moreover, TGF-β-secreting cells such as TAMs and tumor cells suppress T cells, NK cells, decline DC migration, and promote CD4^+^ T cell differentiation into Th2 and Tregs (73). TAMs also interact with CAFs in the TME which is described in this review later. Finally, M2 TAMs can upregulate the expression of both PD-1 and PD-L1. PD-1^+^ TAMs exhibit a declined phagocytosis due to the inhibitory role of PD-1 expression on TAMs compared to PD-1^−^ TAMs (74). Therefore, checkpoint blockade of PD-1/PD-L1 can improve the phagocytosis function of macrophages. PD-L1 expression on TAMs can participate to control T cell activation in the TME.

## Granulocytes

### Basophils

Basophils differentiate and mature in the bone marrow and leave it to circulate in the blood. They participate in allergic diseases, autoimmunity, parasitic infection and Th2 cell differentiation (75, 76). Basophils mediate B cell proliferation, plasma cell survival, and immunoglobulin production (77). By releasing IL-4, they effect different cell types such as fibroblasts, monocytes, macrophages, B and T cells. However, the role of basophils in cancer and the TME is not entirely understood. Basophils interact with other immune cells; for example, they recruit CD8^+^ T cells to the TME by producing CCL3 and CCL4 (5). They also activate these cells through antigen presentation by MHC class I and costimulatory molecule CD86 (75). Interestingly, basophils may require peptide-MHC class II complexes from dendritic cells via trogocytosis which may explain their involvement in CD4 Th2 response activation (78). It was recently shown that regulatory T cells modulate basophil activation through IL-3 and STAT5 however the activation status of basophils in the TME remains obscure (79). Besides interaction with immune cells basophils may indirectly influence tumor growth by VEGF-A production, a proangiogenic factor (80). They may also worsen symptoms due to ascites in patients with ovarian cancer as VEGF-A increases capillary permeabilization. Indeed, ovarian cancer has an enhanced number of basophils in their ascites while their blood level remained low and similar to that of healthy individuals (81). However, in patients with lung cancer, the basophil density in the TME was low compared with other immune cells (82). The presence of basophils in tumor-draining lymph nodes of patients with pancreatic ductal adenocarcinoma and their low number in the blood of colorectal cancer patients was associated with poor prognosis (83). Nevertheless, data is still scarce on the potential role of basophils in cancer. Studies are warranted to show how basophils directly or indirectly help tumor progression and/or tumor immunity.

### Eosinophils

Originated and matured in the bone marrow, eosinophils migrate into the blood circulation to reach the body tissues and to pursue their functions which involves controlling asthma and other allergic diseases, vasodilation, metabolic homeostasis, tissue repair, anti-bacterial, and anti-parasite activity (84–87). Eosinophils release different products such as reactive oxygen species, growth factors (e.g., VEGF and TGF-β), several cytokines (e.g., IL-2 and TNF-α) and lipid mediators (e.g., eicosanoids) (88–90). Eosinophils contribute to antigen presentation to helper T cells and Th2 polarization (91). Therefore, they can promote T cell proliferation by presenting antigens from bacteria, parasites and viruses. Moreover, eosinophils cross-talk to DCs to regulate them. For example, they provide chemotactic abilities for DCs by releasing neurotoxin and induce DC maturation (92, 93). Furthermore, eosinophils regulate other cell types such as basophils, neutrophils and mast cells (94, 95). Eosinophils are commonly seen in the TME (96, 97). In colorectal cancer, their presence was inversely correlated with tumor stage (98). Further, it was shown *in vitro* that eosinophils activated by IFN-γ were capable of killing colorectal cancer cell lines due to their increased cytotoxicity (98). However, regulatory eosinophils, like other regulatory immune cells, promote tumor progression, and suppress immune cells. For example, eosinophils upregulate PD-L1 expression in an IFN-γ-dependent fashion leading to suppression of Th1 responses (99). They can also suppress T cells via galectin-10 by yet not fully understood mechanisms (100). Further by releasing cationic proteins they can hinder antibody production of B cells, inhibit T cell proliferation and elicit mast cell degranulation (101). Eosinophilia has been associated with either poor or better prognosis in different cancers (96, 97). Clearly, characterization of tumor-associated eosinophils and the conditions that promote either pro- or anti-tumorigenic activities is needed to better understand their role in cancer.

### Tumor-Associated Neutrophils

There are two phenotypes of neutrophils known as TANs; anti-tumorigenic N1 neutrophils and pro-tumorigenic N2 neutrophils. Neutrophils type N1 are the first leukocytes to be recruited in the injured/inflamed sites. It was demonstrated that bone marrow neutrophils in mice had higher migration to the inflammation sites in early-stage cancer unlike those from late stage (102). This pattern is mediated by autocrine ATP signaling (102); however, Myh9 has also role in the migration process (103). N1 TANs educate effector T cells to reject tumors, elicit apoptosis of malignant cells by TRAIL, produce and secrete reaction oxygen species (ROS) to lyse cancer cells, participate in ADCC, and release matrix metalloproteinase 8 (MMP-8) to degrade the extracellular matrix (ECM) which is beneficial for tumor metastasis. Nevertheless, N2 TANs have a critical function in the immunosuppression of the TME as the tumor evolves. Blood neutrophilia is noted in patients with advanced cancer. Neutrophilia is stimulated by cytokines such as IL-1β, IL-6, G-CSF, and VEGF secreted by stroma cells and tumors (104). In fact, neutrophils extravasate the blood and recruit into tumor tissue in response to cytokines (IFN-γ and TNF-α), chemokines (KC/CXCL1 and MIP2α/CXCL2 in mice) and cell adhesion molecules (i.e., CD11b) (104). Once infiltrated, TGF-β-stimulated N2 TANs secrete different molecules that shape the TME. They promote angiogenesis via VEGF, promote tumor development and metastasis through cathepsin G and ROS, induce chronic inflammation by releasing TNF-α, IL-1β, IL-6, and IL-12, and inhibit effector CD8^+^ T cells (105). It has been shown that in mice with depleted CD11b^+^/Ly6G^+^ TANs had reduced tumor growth and increased activation of CD8^+^ T cells (106). Moreover, neutrophil-derived leukotrienes selectively expand tumor cells (107). N2 TANs also indirectly preserve tumor cells through recruiting macrophages and Treg cells into the TME (108). Furthermore, TANs were showed to be associated with poor prognosis in several cancer types (109, 110). In addition, enhanced number of CCL2^+^ or CCL17^+^ TANs was correlated with shorter survival rate in patients with hepatocellular carcinoma (108). Due to the abundance of neutrophils in blood and their immediate reaction to tissue injury or inflammation, they are likely important to set the tone of the immune milieu that favors Th2, M2, and N2 responses over Th1-mediated anti-tumor immunity.

## Myeloid-Derived Suppressor Cells

MDSCs are a heterogeneous and commonly immature population of myeloid cells originating from the bone marrow. They share morphology with granulocytes or monocytes. Therefore, the major two MDSC populations are called granulocytic MDSCs (G-MDSC) and monocytic MDSCs (M-MDSC), respectively. MDSCs include myeloid progenitor cells and immature myeloid cells (IMCs). The latest population normally differentiates into mature macrophages, DCs and granulocytes in the peripheral organs in response to growth factors and cytokines (111). Although IMCs in healthy individuals lack suppressive properties, they gain immunosuppressive capability in patients with either cancer or non-neoplastic diseases after activation (111). In the TME, MDSCs are activated by various molecular factors such as VEGF, GM-CSF, C5a, MMP9, IFN-γ, TGF-β, IL-1β, IL-6, IL-10, IL-12, IL-13, CCL2, CXCL5, CXCL12, gangliosides, and prostaglandins (111). As a result of this activation, MDSCs upregulate immune-inhibiting molecules such as Arg1 and inducible nitric oxide synthase (iNOS) (111). The iNOS in turn generates nitric oxide (NO), a product that reduces ADCC in NK cells. Tumor cells secrete CXCL1 to recruit MDSCs into the TME in which they suppress T cell infiltration (16). They highly express indoleamine 2, 3-dioxygenase (IDO) in the TME which results in the stimulation of apoptosis in T cells which was shown in patients with breast cancer (112). IDO can also downmodulates NCRs and DNAM-1 which reduce NK cell-mediated tumor killing. MDSCs also overexpress peroxynitrite (ONOO^−^) which is a strong oxidant that inhibits T cell function (111). G-MDSCs inhibit CD8^+^ T cells through releasing ROS while M-MDSCs do it through secreting Arg1, iNOS, and ROS culminating in the impairment of ζ-chain expression, suppression of MHC class II expression, eliciting T-cell apoptosis, inhibition of Janus kinase (JAK) 3, and STAT5 in which all promote tumor progression (113). Additionally, MDSCs stimulate CD4^+^ T-cell tolerance in a MHC class II-dependent manner (113). Moreover, MDSCs also inhibit immune cell responsiveness to IFNs via reduction of STAT1 phosphorylation in tumor-bearing mice (114). MDSCs may regulate B cell responses since it was shown that the reduction of B cells in the bone marrow of mice with lung cancer was associated with MDSCs infiltration into the TME. Such a regulation was mediated through IL-7 and enhanced activation of STAT5 (115). Furthermore, Tregs are stimulated by MDSCs in a process that requires tumor-associated antigens, IL-10, IFN-γ, and CTLA-4 (111). MDSCs can also be controlled and modulated by tumor cells and other immune cells. For example, tumor cells release exosomes expressing TGF-β and prostaglandin E2 (PGE2) to elicit MDSCs for promoting tumor progression (116). In this *in vivo* experiment, tumor-derived exosomes injected in mice recruited CD11b^+^ Gr-1^+^ cells to tumor and were taken up by them (116). It has been indicated that Tregs in inflammatory conditions enhance MDSC differentiation and function via releasing TGF-β leading to the enhancement of inhibitory functions of MDSCs (117). This cytokine enhances the inhibitory function of the MDSCs. Moreover, mast cells in the TME release histamine that binds to the histamine receptor 1 on MDSCs culminating in the elevation of Arg1 and iNOS and, thus, inhibition of T-cell proliferation (118). MDSCs are multi-potent immunosuppressive cells and, hence, one of the major obstacles to activate and maintain anti-tumor immunity.

## T Regulatory Cells

T regulatory cells significantly infiltrate the tumor and are associated with a poor survival rate (119). Tregs commonly differentiate from naïve CD4^+^ T cells in response to IDO, IL-10, and TGF-β differentiate into CD25^+^ Foxp3^+^ Tregs (called natural Tregs) in the thymus, and into CD25^−^ Tregs including IL-10^+^ Tr1, TGF-β^+^ Th3, and Foxp3^+^ cells (called inducible Tregs or iTregs) in the periphery (120). Thymic mature Tregs originate from CD25^+^ Treg cell progenitors and Foxp3^lo^ Treg cell progenitors (121). Tregs such as NKT Tregs, Qa-1-specific CD8^+^ Tregs, γδ-TCR Tregs, CD8^+^CD28^−^ Tregs, and CD8^+^CD25^+^ Tregs may originate from CD8^+^ T cells as well (122). Resting Tregs are not immunosuppressive unless they become activated through TCR engagement and signaling molecules (123, 124). Tregs exhibit either positive or negative functions depending on conditions such as autoimmunity, infection, pregnancy, inflammation, and cancer. Their positive roles include participation in the immune tolerance, preventing autoimmune diseases, inhibition of tissue damage, and controlling inflammation after infection while their negative role is to hamper cancer immunity. Tregs infiltrate into the TME by signaling molecules such as chemokines and their receptor (e.g., CCL1-CCR8, CCL5-CCR5, CCL22-CCR4, CCL28-CCR10, and CXCL12-CXCR4) (125). After recruitment, Tregs promote tumor growth and metastasis. Tregs release immunosuppressive cytokines including TGF-β, IL-10, and IL-35 which are involved in many of the Treg suppressive functions. For example, DCs maturation is inhibited by TGF-β and IL-10 which can also differentiate them into tDCs (126). Further, it was recently indicated that Tregs debilitate DCs capacity as APCs by removing their peptide-MHC class II complex (127). Hence, Tregs indirectly hamper T cell activation by reducing the number of matured DCs and converting the DCs into tolerogenic partners. However, Tregs directly inhibit T cell activation and reduce their proliferative property by releasing soluble CD25 that sequester IL-2, as well as producing IL-10 and TGF-β (128). The T cell metabolism is further interrupted with adenosine and cAMP by CD39- and CD73-expressing Tregs (120). Treg-released IL-35 influence the expression enhancement of several inhibitory receptors such as PD1, LAG3, and TIM3; thus, promoting intratumoral TIL exhaustion (129). In a similar manner to T cell inhibition, Tregs can directly and indirectly suppress NK cell function. It was elucidated that CD4^+^ CD25^+^ Tregs hinder the NKG2D-mediated NK cell cytotoxicity in a TGF-β-dependent manner, regardless of IL-10 (130). Tregs suppress other immune cells such as basophils, eosinophils, and mast cells as well as B-cell activation and proliferation (120, 131). Finally, it has been demonstrated that Tregs release perforin and granzymes upon T cell receptor engagement which can target cell lysis of T cells and APCs (132).

## Cancer-Associated Adipocytes

Adipocytes and other cell types such as pericytes, endothelial cells, monocytes, macrophages, and pluripotent stem cells make up adipose tissue consisting of two main types white and brown (133). Cancer-associated adipocytes (CAA), or so called fat cells or lipocytes, store energy as triacylglycerol and support cancer cells by providing lipids. Moreover, these cells have pivotal roles in tumorigenesis, tumor growth and metastasis by overexpression of pro-inflammatory cytokines (e.g., IL-1β, Il-6, and IL-10), matrix metalloproteinases (e.g., MMP-11), and insulin-like growth factor binding protein-2 (133–135). Additionally, they release molecules that recruit myeloid cells into the TME, to weak their differentiation status toward M2/MDSC, and promote angiogenesis. These molecules include IL-8, CCL2, VEGF, TGF-β, TNF-α, hepatocyte growth factor, plasminogen activator inhibitor-1, cathepsin S, and monocyte chemoattractant protein-1 (MCP1) (136, 137). Thus, CAAs certainly participate to maintain the deleterious immune milieu in the TME that hinder anti-tumor responses and promote tumor progression.

## Tumor-Associated Endothelial Cells

Tumor-associated endothelial cells (TAEC) line blood vessels in the TME. They possess critical functions such as angiogenesis, permeability, regulating blood fluidity, transportation of the immune cells, intravasation and extravasation of tumor cells during metastasis. Cell adhesion molecules such as intracellular adhesion molecule (ICAM), vascular cell adhesion molecule (VCAM) as well as E- and P-selectin expressed on the endothelial cells mediate leukocyte attachment, rolling, and transmigration into tissue. However, these receptors are commonly dysregulated in blood vessels in the TME reducing the TIL infiltration to the tumor bed. Endothelial cells express not only MHC class I but also MHC class II, therefore they can act as non-professional APCs to elicit antigen-experienced T cells but not naïve T cells due to lack of CD80 and CD86 expression (138). In cancer, endothelial cells undergo abnormalities through overexpression of VEGF and aberrant expression of transcription factors (139, 140). Endothelial cells express TRAIL, FASL, PD-L1, and PD-L2 that can support tumor evasion but also inducible T-cell costimulatory ligand (ICOSL), OX40L, CD40 that may otherwise affect immunity (141). It has been indicated in a mouse model of melanoma that T cell infiltration is enhanced by 18-fold in melanoma cells through inhibition of VEGF and consequently overexpression of CXCL10 and CXCL11 in tumor vessels (142). TAECs play a crucial role in tumor progression to support their required nutrients, and the fragile and dysregulated status of TAEC reduces anti-tumor immunity via dysregulated receptors for TIL migration into tissues.

## Tumor-Associated Pericytes

Tumor-associated pericytes (TAPs) wrap around vascular endothelial cells and closely interact each other, both physically (e.g., gap junctions) and paracrine signaling (143). Together with endothelial cells, pericytes effectively participate in angiogenesis. In addition, they maintain blood flow, modulate ECM remodeling, and contribute to the blood-brain barrier in the brain (143). In cancer, pericytes are abnormal with different marker expression and with cytoplasmic projections into the tumor. Aberrant signaling between pericytes and endothelial cells affect angiogenesis and metastasis in cancer which is reviewed elsewhere (144). Pericytes can participate in immunomodulation of the TME. For instance, in response to IFN-γ, they can upregulate MHC class II and a range of cytokines and chemokines to attract lymphocytes (CXCL10) and granulocytes (CXCL8 and CXCL1) to the site of inflammation (145). However, it has been shown that pericytes upregulate PD-L1 during interaction with tumor cells (146). In line with these findings, pericytes activated by murine glioblastoma diminished T cell activation in the TME (147). Thus, TAPs promote tumor progression and may participate to disrupt anti-tumor T cell responses.

## Cancer-Associated Fibroblasts

Fibroblasts are heterogeneous cells that originate from different cell types such as mesenchyme, epithelial cells, mesothelial cells, fibrocytes, endothelial-mesenchymal transition (EndMT) and epithelial-mesenchymal transition (EMT) (148, 149). They have tissue-specific functions and interact with other stromal cells and immune cells via various signaling molecules. In addition to fibrosis and wound healing, fibroblasts participate in the structure of the ECM through synthesizing collagen, fibronectin, and other constituents (148). They also regulate inflammation and the recruitment of the immune cells through growth factors, cytokines, and chemokines. Cancer-associated fibroblasts (CAFs) are derived from different origins: (1) TGF-β-activated resident fibroblasts; (2) EMT; (3) EndMT; (4) smooth muscle actin-upregulated stellate cells; (5) CAAs; or (6) mesenchymal stem cells with TGF-β overexpression or via CXCL16-CXCR6 (150). There is a reciprocal interaction between tumor cells and CAFs in the TME. Cancer cells stimulate the expression of hepatocyte growth factor (HGF) in CAFs through secreting platelet-derived growth factor, IL-1 and basic fibroblast growth factor (151). HGF plays a critical role in anti-apoptosis, anti-inflammation, and angiogenesis which influences the invasive growth of the tumor (152). Furthermore, CAFs support tumor invasion and dissemination by releasing MMPs (e.g., MMP-2 and MMP-9) and help cancer cell proliferation by releasing exosomes, SDF-1, FGF, IL-6, TGF-β, and osteopontin (150, 153). Indeed, it was recently indicated that CAF-derived exosomes highly express miRNA-21, miRNA-143, and miRNA-378e compared with those from normal fibroblasts (154). After exosome uptake, the tumor cells display EMT and an aggressive phenotype (154). It has also been demonstrated that CD10^+^ GPR77^+^ CAFs can induce the enrichment of cancer stem cells and chemoresistance by secreting pro-inflammatory cytokines IL-6 and IL-8 (155). Besides the interaction with tumor cells, CAFs support angiogenesis by secreting VEGF, FGF2, and FGF7 (150). Compared with normal fibroblasts, CAFs highly express adrenomedullin, a vasodilator peptide hormone, which promote angiogenesis and increase cell tolerance to oxidative stress (156).

Interaction between CAFs and other immune cells has been extensively studied. By secreting TGF-β and IL-10, CAFs convert T cells into iTregs, induce tolerogenic DCs, eliminate APCs by reducing signal activity, inhibit T cell and NK cell function, and activate M2 macrophages. Further, monocytes undergo M2 polarization by M-CSF-secreting CAFs (157, 158). In fact, the number of TAMs significantly correlates with a high grade of CAFs (157). CAFs comprise up to 80% of the tumor mass in pancreatic and breast cancers that are both connected to high level of myeloid cell infiltration (159). A subset of CAFs classified as CAF-S1 provides an immunosuppressive microenvironment in the TME by recruiting CD4^+^ CD25^+^ T cells through OX40 ligand, JAM2, PD-L2, and CXCL12 (160). Moreover, CAF-S1 increases Treg differentiation and expresses B7H3, DPP4, and CD73 to enhance the infiltration of CD25^+^ FOXP3^+^ T lymphocytes (160). Hence, CAFs clearly participate to the immune hostile TME besides their major role to support tumor growth and invasion.

## Tumor-associated Extracellular Vesicles

**Figure 3:**
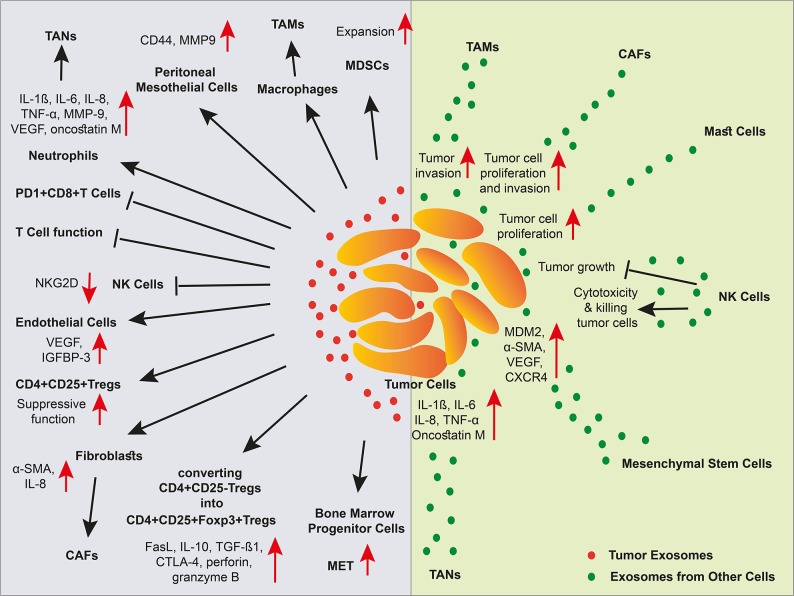
The effect of exosomes in a niche of tumor microenvironment. Different cell types and cancer cells crosstalk in tumor via EVs. In the TME, cancer-associated stroma cells promote tumor progression via exosomes. Tumor cell-derived exosomes inhibit NK cells, and T cells, elicit MDSC expansion and Treg suppressive function, stimulate angiogenesis, and metastasis, and polarize macrophages and neutrophils into TAMs and TANs, respectively. Malignant cells also receive support via exosomes released by cancer-associated stromal cells. However, exosomes released by NK cells induce tumor cell apoptosis through cytotoxicity function.

Almost cells release extracellular vesicles (EVs) such as microvesicles (MVs) and exosomes to communicate with other cells in the body. EVs are taken up by other cells and therefore functioning as a messenger both at short and long distances. The uptake of EVs seems to be selective and depending on the interaction between EV surface proteins with plasma proteins of the recipient cells. For example, it was indicated that milk EVs expressing MUC1, a transmembrane glycoprotein, are taken up by DCs expressing DC-SIGN, a C-type lectin receptor, whereas EVs released by other sources that lack this protein are not internalized by the DCs (161). Herein, we focus on the exosomes that have gained much interest over the past few years. Indeed, exosomes are cup-shaped nanovesicles with a size range of 30–150 nm and an endosomal origin that cargo proteins, lipids, and RNA species (162). Tumor cells commonly release high amounts of exosomes. In cancer, exosomes are involved in most biological features including angiogenesis, immune suppression, metastasis, anti-inflammatory function, anti-apoptosis, drug resistance, and reprogramming the recipient cells via transferring RNA species such as miRNAs. The functions of tumor-derived exosomes on immune cells as well as the immune cell-derived exosomes on tumor cells are summarized in Figure 3.

Metastasis and tumor progression is a hallmark of cancer and can be partly induced by tumor exosomes. Tyrosine protein kinase Met is a hepatocyte growth factor receptor (HGFR) and its activation by HGF triggers the downstream pathways such as Ras, PI3K/STAT3, and β-catenin (163). This oncoprotein is overexpressed in bone marrow progenitor cells by exosomes from melanoma cells which in turn promotes tumor progression (164). Human peritoneal mesothelial cells (HPMCs) uptake exosomes containing CD44 that are released from ovarian cancer cells; therefore, CD44 is elevated in the HPMCs and induced MMP9 secretion which promote tumor invasion (165). Furthermore, metastasis can be promoted by exosomes expressing CD151 and tetraspanin 8 released from rat pancreatic adenocarcinoma cells (166). Both CD151 and tetraspanin 8 contribute to matrix degradation since they are involved in integrin and protease interactions. In a recent study, the effect of tumor exosomes on fibroblasts was revealed. Exosomes released from both early and late stage of colorectal cancer differently activated fibroblasts which led to their conversion into distinct CAF phenotypes. One CAF phenotype formed after induction of early-stage cancer exosomes that had high pro-angiogenic and pro-proliferative properties, and another CAF phenotype formed after stimulation of last-stage cancer exosomes that displayed invasive features (167). Further, CAFs supported tumor cell proliferation and invasion by their exosomes through Wnt-planar cell mobility signaling (168). In chronic myeloid leukemia, tumor cells stimulate bone marrow stromal cells by releasing exosomes (169). After induction, bone marrow stromal cells produce and secrete IL-8 in the TME; thus, leading to the activation of two chemokine receptors, CXCR1 and CXCR2, to promote tumor growth (169). Mesenchymal stem cell (MSC)-derived exosomes commonly lead to tumor growth inhibition (170). However, it has also been shown that exosomes released from MSCs transfer mRNA encoding *CXCR4, VEGF*, α*-SMA*, and *MDM2* to tumor cells, thus enhancing angiogenic activity and tumor growth (171). Furthermore, interaction of cholangiocarcinoma cell-derived EVs with MSCs induces the expression of α-smooth muscle actin, CCL2, CXCL-1, IL-6 in the MSCs which in turn influence the proliferation of tumor cells via enhanced STAT3 phosphorylation (172). Tumor exosomes promote angiogenesis by delivering angiogenic factors into endothelial cells or induction of stroma cells, to support tumor progression. For example, it was described that rat adenocarcinoma tetraspanin-8-expressing exosomes bind to and taken up by endothelial cells which induced the modulation of angiogenic genes in the recipient cells in an VEGF-independent manner (173). Further, treatment of mesenchymal stem cells with exosomes from prostate cancer cells generated myofibroblasts with pro-angiogenic function due to high levels of VEGF-A and pro-invasive property because of high production of MMP−1,−3, and−13 (174). A more recently study showed that exosomes derived from head and neck squamous cell carcinoma were internalized by human umbilical vein endothelial cells (HUVECs) and enhanced vascularization (175). Compared to both pre- and non-malignant cells, exosomes from malignant breast cancer cells containing higher level of annexin II were shown to be very aggressive due to cell mobility (176). Incubation of these exosomes with HUVECs significantly provoked higher angiogenesis (176). In addition, tumor cell-derived EVs transfer membrane-bound epidermal growth factor receptor (EGFR) to endothelial cells. EGFR activates the autocrine vascular growth factor receptor (VEGF)/VEGFR-2 pathway in endothelial cells that supports angiogenesis (177). MVs from other cell types can also contribute to the induction of angiogenesis. For example, exosomes released from bone marrow mesenchymal stem cells can be internalized in tumor cells and elicit angiogenesis through transferring *VEGF, CXCR4*, α*-SMA*, and *MDM2* mRNA transcripts (171).

Immune cells also release exosomes that can affect the tumor or the anti-tumor immune response. NK cell-derived exosomes contain FasL, granulysin, perforin, granzyme A and B therefore exerting cytotoxic activity against tumor cells (178–180). This property is likely true for tumor-targeting T cells as well. For example, it was recently shown that engineered chimeric antigen receptor (CAR) T cells release CAR^+^ exosomes that were cytotoxic to tumor cells (181). Further, it has been indicated that CTL-derived exosomes can provide additional stimulation of bystander CTLs that were previously activated by low affinity antigens (182). Nevertheless, immunosuppression and tumor evasion from the immune recognition in cancer patients are also orchestrated by tumor-derived exosomes and larger released vesicles. For example, it was elucidated that NKG2DL-expressing exosomes from ovarian cancer cells bind to the NKG2D receptor on NK cells, culminating in the NKG2D downregulation as well as impairment of the NK cell-mediated degranulation and cytotoxicity (39). Other studies also showed that tumor exosomes diminish the expression of NKG2D and hamper NK cell cytolytic activity (183). Inhibition of NK cell function is also conducted by hypoxic tumor-derived MVs containing TGF-β1 following their uptake by NK cells and consequently the down-modulation of NKG2D (184). Tumor exosomes can also affect the function of effector CD8^+^ T cells. Hepatocellular cancer cell-derived exosomes can be internalized into the recruited T cells to deliver 14-3-3ζ. The subsequent upregulation of this protein in the T cells leads to the modulation of multiple cellular pathways and down-modulation of their anti-tumor activity (185). Another study showed the influence of phosphatidylserine-expressing EVs from tumor cells on the T cell impairment via the regulation of Akt phosphorylation (186). Moreover, exosomes displaying CD39 and CD73 released from tumor cells could generate adenosine to eliminate T cells (187). The other important role of tumor exosomes is to suppress the function of effector T cells by PD-L1/PD-1 interaction. Tumor exosomes can display PD-L1 and bind to PD-1-expressing effector T cells and suppress their activity even in the draining lymph node demonstrating the long distance communication capacity of tumor cells via the release of exosomes (188). Other effects on the immune system includes the ability of tumor exosomes to reduce ADCC by binding tumor-reactive antibodies that would otherwise target the tumor cells (189). Furthermore, exosomes released from gastric cancer cells induced N2 polarization of TANs and these cells promoted cancer cell migration (190). They can also elicit MDSC expansion by STAT3 activation via membrane-associated Hsp72, and recruit Tregs expressing CCL20 into the TME (191, 192). Tumor-derived exosomes were shown to mediate the conversion of CD4^+^ CD25^−^ T cells into CD4^+^ CD25^high^ FOXP3^+^ regulatory T cells with enhanced expression of Fas ligand, IL-10, TGF-β1, CTLA-4, granzyme B, and perforin (193). Moreover, the effect of tumor exosomes in macrophage polarization has been shown in recent studies. Tumor-derived exosomes stimulate macrophage polarization into M2 phenotype following activation of NF-κB pathway and enhanced gene expression of pro-inflammatory cytokines such as *IL-1*β and *IL-8* (194). Such a macrophage polarization was also shown by hypoxic tumor exosomes enriched in CCL2, CSF1, TGF-β, macrophage migration inhibitory factor (MIF), ferritin heavy/light chain, endothelial monocyte-activating polypeptide 2 (EMAP2), and leukotriene A-4 hydrolase (195).

The impact of tumor EVs on their targets is not limited to their protein content since they also convey genetic materials such as miRNA and lncRNA transcripts that can profoundly affect the targets. The EVs from other cell origins also contribute to tumorigenesis due to their RNA species. It has explicitly been elucidated that miRNAs possess diverse expression and play critical functions in the body. In general, miRNAs implicate in cell development, proliferation, differentiation, apoptosis, and diseases including cancer. Both endogenous and exogenous miRNAs are packed into the EVs. These miRNAs like other RNA species are preserved in exosomes from enzymatic degradation (196). Exosomal miRNAs are delivered into recipient cells to execute functional roles in the targets and examples of these roles are described elsewhere (197). Briefly, such functions include: (1) contribution to chemotherapy resistance via miR-21 and miR-155 (198); (2) provoking angiogenesis in endothelial cells with miR-210 (199); (3) NK cell inhibition by miR-23a through targeting CD107a (184); (4) T cell elimination via miR-24-3p (200); (5) inducing polarization of macrophages into M2 phenotype via miR-222-3p, miR-940 and miR-301a (201–203); and (6) promoting tumor cell proliferation and metastasis via miR-148a and miR-423-5p (204, 205). However, exosomes released by stromal cells such as TAMs and CAFs can elicit tumor invasion and metastasis via miR-223, miR-21, miR-143, and miR-378e (154, 206). In addition to miRNA transcripts, lncRNAs carried by tumor-derived MVs and exosomes can be associated with tumor progression. Cancer cells release H19-containing exosomes that modulate endothelial cells to enhance angiogenesis (207). Hypoxic exosomes from bladder cancer cells carry urothelial cancer-associated 1 (UCA1) that augment tumor growth through epithelial-mesenchymal transition (208). Metastasis-associated lung adenocarcinoma transcript 1 (MALA1) is another lncRNA that can be sorted into exosomes of epithelial ovarian cancer cells and shuttled into HUVECs and increase angiogenesis (209). In a study, the induction of tumor invasion and migration was mediated by exosomal HOX transcript antisense RNA (HOTAIR) originated from bladder cancer cells (210).

## Metabolites

Metabolism is biochemical reactions in the organism, transforming one chemical into another via metabolic pathways. Metabolism is ensuring that the food we eat will be converted to sources of energy and building blocks for DNA-, protein-, and fat synthesis. During malignancy, the cell metabolism undergoes changes to support the progressing tumor and the malignant phenotype (211). For example, due to hypoxia and acidic conditions the tumor switches to glycolytic metabolism which further reduces pH due to lactic acid production by the tumor as reviewed elsewhere (212). The immune system is affected by the metabolic alterations in the TME. For example, TILs react to high acidic condition, the absence of oxygen and the unfavorable composition of metabolites in the TME. While a low pH can trigger maturation of DCs likely due to protons being recognized by DAMP, T cells will become less prone to cytolytic responses and IFN-γ production. However, the T cell anergy can be reversed if the T cells are exposed to normal conditions (213, 214). Hypoxia plays a crucial role in drug resistance, angiogenesis, stimulation of metastasis, and tumor invasion as well as immune suppression (215, 216). It has also been shown that hypoxia contributes to an immunosuppressive microenvironment through upregulation of checkpoint molecules including modulation of CD47, HLA-G, and PD-L1, and finally regulation of lactate and adenosine (216). A hypoxic microenvironment together with lactic acid reduces T cell activation, proliferation and lytic ability (215). Hypoxia also attracts TAMs, Treg cells, and MDSCs into the TME (215). Different metabolites also affect the immune system in the TEM including arginase, adenosine, IDO, and NOS. Their actions are briefly discussed herein.

Arginine known as L-arginine (L-Arg) is an essential α-amino acid which is beneficial for the immune cells. For example, L-Arg is required for the expression of the CD3ζ chain and therefore crucial for TCR signaling and cytotoxicity (217, 218). Nevertheless, L-Arg might be depleted by L-Arg-degrading enzymes such as arginases. Such a depletion is conducted by TAMs, N2 neutrophils and monocytic MDSCs that release high level of arginase in the TME which subsequently metabolizes L-Arg into urea and L-ornithine (219). It was shown *in vivo* that L-Arg depletion accumulates MDSCs and prevents T cell proliferation (220). Interestingly, arginase inhibitors partially eliminate MDSCs which produce high levels of arginase *ex vivo* and can impede myeloid cell-mediated suppression in the TME and enhance TIL function (221, 222).

The cellular metabolite adenosine is a nucleoside derived from ATP barely present in the extracellular space in healthy tissue. However, upon injury it is present due to an ATP dephosphorylation process leading to adenosine. In the extracellular space adenosine binds to its cellular receptors. Adenosine is important in diverse functions, reviewed elsewhere (223) but in the TME it is involved in immunosuppression. High adenosine concentration suppresses CTLs and NK cells by diminishing their cytotoxic capabilities because of a cAMP build-up which leads to protein kinase A (PKA) activity and thereby a negative effect on signaling pathways needed for T cell and NK cell function (223, 224). The NK cell maturation is also inhibited by adenosine (225). Blocking adenosine is illustrated elsewhere (223).

Three enzymes IDO1, IDO2, and tryptophan-2,3-dioxygenase (TDO) catabolize tryptophan (Trp), an essential amino acid for physiological processes such as immune regulation, into kynurenine (kyn). IDO1 is produced in mesenchymal stromal cells, endothelial cells, fibroblasts, tolerogenic DCs, TAMs, CAFs, MDSCs, and elevated in tumor cells and can be loaded into tumor-derived microvesicles (226–228). IDO1 elevation has pivotal roles in tumorigenesis and immunosuppression, resistance to chemotherapies and is correlated to shorter survival in cancer patients (229–231). The immunosuppressive IDO halts NK cell cytotoxicity, inhibits the infiltration and function of CD8^+^ T cells, recruits and activates MDSCs and Tregs in the TME, stimulates differentiation of CD4^+^ T cells into Treg phenotypes through activation of AhR by Kyn, induces tumor tolerance to apoptosis, impairs TCR via Vav1 elimination, elicits differentiation of immunogenic DCs into tolerogenic DCs, stimulates polarization of macrophages into TAMs, exhorts resistance to checkpoint inhibitors, and suppresses CD19-CART cells (228, 232–238). Moreover, IDO influences PD-1 overexpression by Tregs and these cells in turn stimulate expression of PD-L1 and PD-L2 on DCs (235). Such a process negatively modulates T cells. However, blocking these ligands by monoclonal antibodies enhances T-cell proliferation and activation (239). Since IDO elevation is associated with carcinogenesis and tumor progression, there has been efforts to inhibit this enzyme clinically. Different strategies have been applied for cancer patients regarding IDO pathway; such as inhibition of IDO1, AhR, Kyn, Trp-Kyn pathway and dual IDO1-TDO inhibition. Although IDO1 inhibitors showed promising results in clinical trials, a combination of Epacadostat, an IDO1 inhibitor, with pembrolizumab, a PD-1 checkpoint inhibitor, did not show a significant difference with placebo plus pembrolizumab (240).

NOS is an enzyme catalyzing the production of nitric oxide (NO) from the metabolite L-arginine. An inducible form, iNOS, is commonly expressed in cancer which leads to high NO levels which can caser DNA damage, oncogene activation, inhibit DNA repair enzymes and suppressor genes as reviewed elsewhere (241). However, NOS functions as either anti-tumor or pro-tumor, depending on its concentration (242). At high concentration, NO enhances the anti-tumor activity through broad DNA damage, cell apoptosis, cytotoxicity, oxidative, or nitrosative stress whereas at low concentration, it increases cell proliferation, apoptosis, angiogenesis, invasiveness, and metastasis (242). In addition to cancer cells; macrophages, neutrophils, hepatocytes, endothelial cells, cardiomyocytes, and chondrocytes can produce high levels of NO (219). Interaction between NO and reactive oxygen species forms nitrogen dioxide, reactive nitrogen species and peroxynitrite (ONOO-) in which the latest product stimulates carcinogenesis such as angiogenesis, metastasis, apoptosis elimination, DNA damage induction, and increasing cell proliferation; however, it may acts as an antioxidant (242). Hence, this ion play important roles under pathophysiological conditions. Of interest, peroxynitrite can nitrate both subunits α and β of the CD8 T cell receptor thus impairing antigen recognition (243). In addition, MDSC-released peroxynitrite inhibits the recruitment of effector CD8^+^ T cells via nitration of CCL2 (244). Further, iNOS upregulation is elicited by a cytokines secreted by Th1 cells (e.g., IL-1, TNF-α, and IFN-γ) as well as hypoxia and lipopolysaccharide (219). Hence, these events augment the deficiency of L-Arg which in turn provides immunosuppression and tumor progression (219). In terms of tumor eradication, NOS inhibitors have been used in colorectal cancer to eliminate the migration of malignant cells by interruption of angiogenesis pathway (245).

## Immunotherapy—the Challenges of the TME

Activation of the immune system to combat cancer has been an appealing treatment method that for many decades eluded success in the vast majority of cancer indications. However, immunotherapy in the form of bacillus Calmette-Guerin (BCG) has been standard-of-care for non-muscle-invasive urinary bladder cancer since the past 40 years (246). These patients have rather low tumor burden at the stages when BCG is successful, meaning that the immunosuppressive mechanisms are still not advanced. With the growing body-of-evidence pin-pointing the immune escape mechanisms used by the tumor and its microenvironment, it has become evident that these mechanisms need to be combated to fully activate antitumor immunity. The first attempts were performed by Rosenberg et al., whom combined T cell therapy with so called preconditioning. The preconditioning consisted of cyclophosphamide and fludarabine given a few days prior to infusion of *ex vivo*-stimulated TILs to reduce regulatory T cells. This strategy, sometimes combined with whole body irradiation, finally resulted in clinical responses to immunotherapy in patients with melanoma (247). This was the first study demonstrating that immunotherapy could eradicate solid cancer in last stage patients and these pivotal steps built the basis for T cell therapy including the successful protocols using genetically engineered CAR T cells (248, 249). The increasing understanding of how T cells are regulated revealed that after activation, T cells upregulate checkpoint receptors that initiate self-regulation upon stimulation by counter receptors. Such checkpoints including PD-1 and CTLA-4 are important to restrict unwanted immunity as well as to contract responses when after an infection is eradicated. However, stimulation of checkpoint receptors in cancer is commonly chronic and lead to a premature abruption of the immune response. Antibodies blocking CTLA-4, PD-1, or the PD-L1 counter receptor are collectively called checkpoint inhibitors (CPI).

CPIs has changed the cancer therapeutic landscape over the past few years and have added immunotherapy to the cornerstones of cancer therapeutics (250). For many indications such as melanoma and lung cancer, a CPI is now the first line treatment over conventional chemotherapy and radiotherapy. Although CPI has shown impressive outcomes, especially in immune sensitive tumors, the majority of patients are refractory or become resistant (251). Such mechanisms of resistance were recently reviewed elsewhere (252). Briefly, unresponsive patients commonly have a poor TIL level in the tumor which may depend on few neoantigens leading to robust T cell responses. For example, it is known that patients with mismatch repair defects have a high level of mutations and consequently a wide range of antigens for TILs to target. These patients have a high response rate to CPI while those lacking mismatch repair defects commonly have a poor outcome (253). Another resistance mechanism in refractory patients is a defect antigen-presentation machinery. Such defects include β2-microglobulin (B2M) deficiency leading to absence of MHC class-I on the cell surface or defects in the antigen processing machinery (254). Hence, these defected cells are invisible to T cell recognition. However, besides intrinsic tumor resistance, the TME including immunosuppressive immune cells certainly contribute to hamper immunotherapy such as CPI. Likely, any therapy aiming to reduce immunosuppression, like CPI, must be given simultaneously to other agents that activate immune reactions or counteract other suppressive arms such as the myeloid cell compartment.

Immune activating therapies include a wide range of different treatment modalities such as tumor-peptide-based vaccines, oncolytic viruses, agonistic antibodies and a variety of immune cell therapies based on T cells, NK cells, and dendritic cells reviewed elsewhere (255). Options to reduce the action of immunosuppressive cells or molecules are also being evaluated. For example, antibodies that blocks chemokine receptors may prevent accumulation of suppressive myeloid cells such as M2 macrophages in the TME (256), the small-molecule mebendazole polarizes macrophages toward the anti-tumoral M1 phenotype (257) and common chemotherapeutics such as gemcitabine, or tyrosine kinase inhibitors, reduces both MDSCs and/or Tregs in patients (258–260). Such agents may be of high interest to combine with CPIs and activating immunotherapeutics. However, agents affecting angiogenesis may be of interest since they not only limit blood supply to the tumors but also normalize the otherwise dysregulated blood vessels in the tumor tissue. Such a normalization may allow for better lymphocyte attachment, rolling, and transmigration into the tumor site (261). Table 1 shows a selection of clinical trials that correlated clinical observations after treatment with different immunostimulatory/TME modulating agents to the level of immune cell findings in the tumor or blood.

**Table 1:** Clinical outcomes correlated with the cell density alteration of cancer patients after immunotherapies.

**Cancer type**	**Treatment**	**Cell types**	**Clinical correlation**	**References**
Prostate	Ipilimumab	CTL ↑ (*p* < 0.05) Memory T cells ↑ (*p* < 0.05)	OS ↑ PFS ↑	(262)
NSCLC	Nivolumab Pembrolizumab	CTL ↑ (*p* < 0.05) ND	OS ↑ PFS ↑ RR ↑ ORR ↑ PFS ↑	(263) (264)
Urothelial	Atezolizumab	CTL ↑ (*p* = 0.02)	OS ↑ ORR ↑	(265)
Melanoma	AdCD40L Pembrolizumab Nivolumab Nivolumab Pembrolizumab + T-VEC Ipilimumab + ATRA Ipilimumab	CTL ↑ (*p* = 0.0001) CTL ↑ (*p* < 0.0001) ND Th9 cells ↑ (*p* < 0.05) Th2 ↑ (*p* < 0.05) Memory T ↑ (*p* < 0.001) B cells ↑ (*p* < 0.001) CD4^+^ T ↑ (*p* < 0.001) CD68^+^ cells ↑ (*p* < 0.001) CTL ↑ (*p* < 0.001) Treg ↓ (*p* < 0.001) MDSCs ↓ (*p* < 0.05) CD4^+^ ICOS^+^ T ↑*P* < 0.0001 Treg ↓ (*p* < 0.001) EM CD8^+^ T ↑ MoMDSCs ↓ (*p* < 0.001)	NS OS ↑ PFS ↑ ND OS & PFS (NS) OS & PFS (NS) OS & PFS (NS) OS & PFS (NS) OS & PFS (NS) OS & PFS (NS) ND	(266) (267) (268) (269) (270) (271) (272)
Bladder	AdCD40L	Treg ↓ (*p* = 0.008) CTL ↓ (*p* = 0.02)	ND	(273)
Renal	IL-2–based immunotherapy	Treg ↑ (*p* < 0.001) Neutrophils ↓ (*p* = 0.026) CD3^+^ cells ↑ (*p* = 0.002) CD8^+^ T ↑ (*p* = 0.003) CD57^+^ NK ↑ (*p* = 0.001)	Poor prognosis PR ↑ PR ↑ PR ↑	(274, 275)
Lung, hepatic	NK cell- immunotherapy	NK cells ↑ (*p* < 0.01) CD3^+^ T cells (*p* < 0.01)	PFS ↑	(276, 277)
Gastric	RAM-containing chemotherapy	PD-1^+^ CD8^+^ T cells ↓ Treg ↓*P* = 0.034	NS NS	(278)
Colorectal	Bevacizumab plus chemotherapy	Treg ↓ (*P* = 0.0039)	ND	(279)
ALL, lymphoma	CAR T cells	MDSCs ↓ (*p* = ?)	OS ↑	(280)
Glioblastoma	Bevacizumab	Treg ↓ (*p* < 0.01) Neutrophils (*p* < 0.05) Monocytes (*p* < 0.001)	OS ↓ OS ↑ OS ↑	(281)
HNC	Tadalafil + M/pICLC V	MDSCs ↓ blood (*p* < 0.05) Treg ↓ blood (*p* < 0.018) MDSCs ↓ tissue, *p* = 0.007 Treg ↓ tissue, *p* = 0.007 CD8^+^ T ↑ tissue, *p* = 0.023	ND ND ND ND OS ↑	(282)

*Clinical correlations were statistically significant unless mentioned. NSCLC, non–small-cell lung cancer; HNC, head and neck carcinoma; ALL, acute lymphoblastic leukemia; T-VEC, Talimogene Laherparepvec; RAM, Ramucirumab- containing chemotherapy; M/pICLC V, MUC1/polyICLC vaccine; ATRA, all-trans retinoic acid; EM, effector memory; ND, not determined; OS, overall survival; ORR, objective response rate; SD, stable disease; PR, partial response; PFS, progression-free survival; ORR, overall response rate; CRR, complete response rate; RR, response rate; NS, not significant*.

## Concluding Remarks

The TME is clearly complex and participates to promote tumor formation, progression, and metastasis while hampering anti-tumor immunity. It is important to consider the different aspects of the TME when designing new therapies and new treatment protocols for cancer patients. Many activating immunotherapies have failed to show clinical benefit over the years which is likely due to the previous lack of understanding of immunosuppression in the patients. As CPIs entered the stage, it is now possible to release the TME break of CTLs but CPI therapy is dependent on *de novo* T cell activation. Hence, combinations of an immune activating treatment and CPIs are appealing. However, active immune cells must be able to enter into the tumor parenchyma to exert their function. The next generation of immunotherapy trials will likely involve drugs that can normalize tumor blood vessels. Moreover, considering the highly immunosuppressive role of fibroblasts and other stroma, novel treatments that may target the function of these cells should be considered together with immunotherapy. Finally, more efforts need to consider microbiota and lifestyle mechanisms in the efficacy evaluation of immunotherapy.

## Author Contributions

Conception of idea was from AL-M. All contributed to the writing of the review. AL-M the most and MA-M the least. AL-M prepared the figures and the table. AL supervised and edited the manuscript. All authors approved the final version of the manuscript.

## Conflict of Interest

AL declares the following conflict of interests: she is the CEO and board member of Lokon Pharma AB, the chairman of the board of Repos Pharma AB and Vivolux AB and a board member of Bioimics AB. She is a alternate board member and advisor to NEXTTOBE AB. She also holds a contract research agreement with Lokon Pharma AB and has royalty agreements with Lokon Pharma AB and Alligator Biosciences AB. The remaining authors declare that the research was conducted in the absence of any commercial or financial relationships that could be construed as a potential conflict of interest.

## References

[1] Swann JB Smyth MJ. Immune surveillance of tumors. J Clin Investig. (2007) 117:1137–46. 10.1172/JCI31405 17476343 PMC1857231

[2] Gül N Babes L Siegmund K Korthouwer R Bögels M Braster R. Macrophages eliminate circulating tumor cells after monoclonal antibody therapy. J Clin Investig. (2014) 124:812–23. 10.1172/JCI66776 24430180 PMC3904600

[3] Martin JH Edwards SW. Changes in mechanisms of monocyte/macrophage-mediated cytotoxicity during culture. Reactive oxygen intermediates are involved in monocyte-mediated cytotoxicity, whereas reactive nitrogen intermediates are employed by macrophages in tumor cell killing. J Immunol. (1993) 150:3478–86. 8385686

[4] Urban JL Shepard HM Rothstein JL Sugarman BJ Schreiber H. Tumor necrosis factor: a potent effector molecule for tumor cell killing by activated macrophages. Proc Natl Acad Sci USA. (1986) 83:5233–37. 10.1073/pnas.83.14.5233 3487788 PMC323925

[5] Sektioglu IM Carretero R Bulbuc N Bald T Tuting T Rudensky AY. Basophils promote tumor rejection via chemotaxis and infiltration of CD8+ T cells. Cancer Res. (2017) 77:291–302. 10.1158/0008-5472.CAN-16-0993 27879269

[6] Lotfi R Lee JJ Lotze MT. Eosinophilic granulocytes and damage-associated molecular pattern molecules (DAMPs): role in the inflammatory response within tumors. J Immunother. (2007) 30:16–28. 10.1097/01.cji.0000211324.53396.f6 17198080

[7] Kataoka S Konishi Y Nishio Y Fujikawa-Adachi K Tominaga A. Antitumor activity of eosinophils activated by IL-5 and eotaxin against hepatocellular carcinoma. DNA Cell Biol. (2004) 23:549–60. 10.1089/dna.2004.23.549 15383175

[8] Legrand F Driss V Delbeke M Loiseau S Hermann E Dombrowicz D. Human eosinophils exert TNF-alpha and granzyme A-mediated tumoricidal activity toward colon carcinoma cells. J Immunol. (2010) 185:7443–51. 10.4049/jimmunol.1000446 21068403

[9] Hubert M Gobbini E Couillault C Manh T-PV Doffin A-C Berthet J. IFN-III is selectively produced by cDC1 and predicts good clinical outcome in breast cancer. Sci Immunol. (2020) 5:eaav3942. 10.1126/sciimmunol.aav3942 32303573

[10] Li H Shi B. Tolerogenic dendritic cells and their applications in transplantation. Cell Mol Immunol. (2015) 12:24–30. 10.1038/cmi.2014.52 25109681 PMC4654373

[11] Kushwah R Wu J Oliver JR Jiang G Zhang J Siminovitch KA. Uptake of apoptotic DC converts immature DC into tolerogenic DC that induce differentiation of Foxp3+ Treg. Eur J Immunol. (2010) 40:1022–35. 10.1002/eji.200939782 20101618 PMC4603937

[12] Gregori S Tomasoni D Pacciani V Scirpoli M Battaglia M Magnani CF. Differentiation of type 1 T regulatory cells (Tr1) by tolerogenic DC-10 requires the IL-10-dependent ILT4/HLA-G pathway. Blood. (2010) 116:935–44. 10.1182/blood-2009-07-234872 20448110

[13] Ben-Baruch A. Organ selectivity in metastasis: regulation by chemokines and their receptors. Clin Exp Metastasis. (2008) 25:345–56. 10.1007/s10585-007-9097-3 17891505

[14] Hojo S Koizumi K Tsuneyama K Arita Y Cui Z Shinohara K. High-level expression of chemokine CXCL16 by tumor cells correlates with a good prognosis and increased tumor-infiltrating lymphocytes in colorectal cancer. Cancer Res. (2007) 67:4725–31. 10.1158/0008-5472.CAN-06-3424 17510400

[15] Curiel TJ Coukos G Zou L Alvarez X Cheng P Mottram P. Specific recruitment of regulatory T cells in ovarian carcinoma fosters immune privilege and predicts reduced survival. Nat Med. (2004) 10:942–9. 10.1038/nm1093 15322536

[16] Li J Byrne KT Yan F Yamazoe T Chen Z Baslan T. Tumor cell-intrinsic factors underlie heterogeneity of immune cell infiltration and response to immunotherapy. Immunity. (2018) 49:178–93.e7. 10.1016/j.immuni.2018.06.006 29958801 PMC6707727

[17] Gooden MJM de Bock GH Leffers N Daemen T Nijman HW. The prognostic influence of tumour-infiltrating lymphocytes in cancer: a systematic review with meta-analysis. Br J Cancer. (2011) 105:93–103. 10.1038/bjc.2011.189 21629244 PMC3137407

[18] Yao W He JC Yang Y Wang JM Qian YW Yang T. The prognostic value of tumor-infiltrating lymphocytes in hepatocellular carcinoma: a systematic review and meta-analysis. Sci Rep. (2017) 7:7525. 10.1038/s41598-017-08128-1 28790445 PMC5548736

[19] Maimela NR Liu S Zhang Y. Fates of CD8+ T cells in tumor microenvironment. Comput Struct Biotechnol J. (2019) 17:1–13. 10.1016/j.csbj.2018.11.004 30581539 PMC6297055

[20] Dangaj D Bruand M Grimm AJ Ronet C Barras D Duttagupta PA. Cooperation between constitutive and inducible chemokines enables T cell engraftment and immune attack in solid tumors. Cancer Cell. (2019) 35:885–900.e10. 10.1016/j.ccell.2019.05.004 31185212 PMC6961655

[21] De Sousa Linhares A Leitner J Grabmeier-Pfistershammer K Steinberger P. Not all immune checkpoints are created equal. Front Immunol. (2018) 9:1909. 10.3389/fimmu.2018.01909 30233564 PMC6127213

[22] Beatty G Paterson Y. IFN-gamma-dependent inhibition of tumor angiogenesis by tumor-infiltrating CD4+ T cells requires tumor responsiveness to IFN-gamma. J Immunol. (2001) 166:2276–82. 10.4049/jimmunol.166.4.2276 11160282

[23] Hoepner S Loh JM Riccadonna C Derouazi M Maroun CY Dietrich PY. Synergy between CD8 T cells and Th1 or Th2 polarised CD4 T cells for adoptive immunotherapy of brain tumours. PLoS ONE. (2013) 8:e63933. 10.1371/journal.pone.0063933 23717511 PMC3662716

[24] Ellyard JI Simson L Parish CR Th2-mediated anti-tumour immunity: friend or foe? Tissue Antigens. (2007) 70:1–11. 10.1111/j.1399-0039.2007.00869.x 17559575

[25] Bailey SR Nelson MH Himes RA Li Z Mehrotra S Paulos CM. Th17 cells in cancer: the ultimate identity crisis. Front Immunol. (2014) 5:276. 10.3389/fimmu.2014.00276 24987392 PMC4060300

[26] Coussens LM Werb Z. Inflammation and cancer. Nature. (2002) 420:860–7. 10.1038/nature01322 12490959 PMC2803035

[27] Jia L Wu C. The biology and functions of Th22 cells. Adv Exp Med Biol. (2014) 841:209–30. 10.1007/978-94-017-9487-9_8 25261209

[28] Lu Y Wang Q Xue G Bi E Ma X Wang A. Th9 cells represent a unique subset of CD4(+) T cells endowed with the ability to eradicate advanced tumors. Cancer Cell. (2018) 33:1048–60.e7. 10.1016/j.ccell.2018.05.004 29894691 PMC6072282

[29] Micosse C von Meyenn L Steck O Kipfer E Adam C Simillion C. Human “TH9” cells are a subpopulation of PPAR-gamma(+) TH2 cells. Sci Immunol. (2019) 4:eaat5943. 10.1126/sciimmunol.aat5943 30658968

[30] Kaplan MH Hufford MM Olson MR. The development and *in vivo* function of T helper 9 cells. Nat Rev Immunol. (2015) 15:295–307. 10.1038/nri3824 25848755 PMC4445728

[31] Bauer S Groh V Wu J Steinle A Phillips JH Lanier LL. Activation of NK cells and T cells by NKG2D, a receptor for stress-inducible MICA. Science. (1999) 285:727–9. 10.1126/science.285.5428.727 10426993

[32] Eagle RA Jafferji I Barrow AD. Beyond stressed self: evidence for NKG2D ligand expression on healthy cells. Curr Immunol Rev. (2009) 5:22–34. 10.2174/157339509787314369 19626129 PMC2713595

[33] Imai K Matsuyama S Miyake S Suga K Nakachi K. Natural cytotoxic activity of peripheral-blood lymphocytes and cancer incidence: an 11-year follow-up study of a general population. Lancet. (2000) 356:1795–9. 10.1016/S0140-6736(00)03231-1 11117911

[34] Fionda C Malgarini G Soriani A Zingoni A Cecere F Iannitto ML. Inhibition of glycogen synthase kinase-3 increases NKG2D ligand MICA expression and sensitivity to NK cell-mediated cytotoxicity in multiple myeloma cells: role of STAT3. J Immunol. (2013) 190:6662–72. 10.4049/jimmunol.1201426 23686482

[35] Nausch N Cerwenka A. NKG2D ligands in tumor immunity. Oncogene. (2008) 27:5944–58. 10.1038/onc.2008.272 18836475

[36] Salih HR Rammensee HG Steinle A. Cutting edge: down-regulation of MICA on human tumors by proteolytic shedding. J Immunol. (2002) 169:4098–102. 10.4049/jimmunol.169.8.4098 12370336

[37] Stern-Ginossar N Gur C Biton M Horwitz E Elboim M Stanietsky N. Human microRNAs regulate stress-induced immune responses mediated by the receptor NKG2D. Nat Immunol. (2008) 9:1065–73. 10.1038/ni.1642 18677316

[38] Yamada N Yamanegi K Ohyama H Hata M Nakasho K Futani H. Hypoxia downregulates the expression of cell surface MICA without increasing soluble MICA in osteosarcoma cells in a HIF-1alpha-dependent manner. Int J Oncol. (2012) 41:2005–12. 10.3892/ijo.2012.1630 22992985

[39] Labani-Motlagh A Israelsson P Ottander U Lundin E Nagaev I Nagaeva O. Differential expression of ligands for NKG2D and DNAM-1 receptors by epithelial ovarian cancer-derived exosomes and its influence on NK cell cytotoxicity. Tumour Biol. (2016) 37:5455–66. 10.1007/s13277-015-4313-2 26563374

[40] Bruno A Ferlazzo G Albini A Noonan DM. A think tank of TINK/TANKs: tumor-infiltrating/tumor-associated natural killer cells in tumor progression and angiogenesis. J Natl Cancer Inst. (2014) 106:dju200. 10.1093/jnci/dju200 25178695 PMC4344546

[41] Rocca YS Roberti MP Arriaga JM Amat M Bruno L Pampena MB. Altered phenotype in peripheral blood and tumor-associated NK cells from colorectal cancer patients. Innate Immun. (2013) 19:76–85. 10.1177/1753425912453187 22781631

[42] Park YJ Song B Kim YS Kim EK Lee JM Lee GE. Tumor microenvironmental conversion of natural killer cells into myeloid-derived suppressor cells. Cancer Res. (2013) 73:5669–81. 10.1158/0008-5472.CAN-13-0545 23867469

[43] Artis D Spits H. The biology of innate lymphoid cells. Nature. (2015) 517:293–301. 10.1038/nature14189 25592534

[44] Spits H Bernink JH Lanier L. NK cells and type 1 innate lymphoid cells: partners in host defense. Nat Immunol. (2016) 17:758–64. 10.1038/ni.3482 27328005

[45] Irshad S Flores-Borja F Lawler K Monypenny J Evans R Male V. RORgammat(+) innate lymphoid cells promote lymph node metastasis of breast cancers. Cancer Res. (2017) 77:1083–96. 10.1158/0008-5472.CAN-16-0598 28082403

[46] Gao Y Souza-Fonseca-Guimaraes F Bald T Ng SS Young A Ngiow SF. Tumor immunoevasion by the conversion of effector NK cells into type 1 innate lymphoid cells. Nat Immunol. (2017) 18:1004–15. 10.1038/ni.3800 28759001

[47] Balato A Unutmaz D Gaspari AA. Natural killer T cells: an unconventional T-cell subset with diverse effector and regulatory functions. J Invest Dermatol. (2009) 129:1628–42. 10.1038/jid.2009.30 19262602

[48] Metelitsa LS Wu HW Wang H Yang Y Warsi Z Asgharzadeh S. Natural killer T cells infiltrate neuroblastomas expressing the chemokine CCL2. J Exp Med. (2004) 199:1213–21. 10.1084/jem.20031462 15123743 PMC2211904

[49] Tiwary S Berzofsky JA Terabe M. Altered lipid tumor environment and its potential effects on NKT cell function in tumor immunity. Front Immunol. (2019) 10:2187. 10.3389/fimmu.2019.02187 31620124 PMC6759687

[50] Kumar V Delovitch TL. Different subsets of natural killer T cells may vary in their roles in health and disease. Immunology. (2014) 142:321–36. 10.1111/imm.12247 24428389 PMC4080948

[51] Wong CH Kubes P. Imaging natural killer T cells in action. Immunol Cell Biol. (2013) 91:304–10. 10.1038/icb.2013.6 23381461

[52] Renukaradhya GJ Khan MA Vieira M Du W Gervay-Hague J Brutkiewicz RR. Type I NKT cells protect (and type II NKT cells suppress) the host's innate antitumor immune response to a B-cell lymphoma. Blood. (2008) 111:5637–45. 10.1182/blood-2007-05-092866 18417738 PMC2424159

[53] Jiang S Game DS Davies D Lombardi G Lechler RI. Activated CD1d-restricted natural killer T cells secrete IL-2: innate help for CD4+CD25+ regulatory T cells? Eur J Immunol. (2005) 35:1193–200. 10.1002/eji.200425899 15770696

[54] Jung KY Cho SW Kim YA Kim D Oh BC Park DJ. Cancers with higher density of tumor-associated macrophages were associated with poor survival rates. J Pathol Transl Med. (2015) 49:318–24. 10.4132/jptm.2015.06.01 26081823 PMC4508569

[55] Zhang Q-w Liu L Gong C-y Shi H-s Zeng Y-h Wang X-z. Prognostic significance of tumor-associated macrophages in solid tumor: a meta-analysis of the literature. PLoS ONE. (2012) 7:e50946. 10.1371/journal.pone.0050946 23284651 PMC3532403

[56] Allavena P Piemonti L Longoni D Bernasconi S Stoppacciaro A Ruco L. IL-10 prevents the generation of dendritic cells from CD14+ blood monocytes, promotes the differentiation to mature macrophages and stimulates endocytosis of FITC-dextran. Adv Exp Med Biol. (1997) 417:323–7. 10.1007/978-1-4757-9966-8_53 9286381

[57] Chomarat P Banchereau J Davoust J Palucka AK. IL-6 switches the differentiation of monocytes from dendritic cells to macrophages. Nat Immunol. (2000) 1:510–4. 10.1038/82763 11101873

[58] Gabrilovich D Ishida T Oyama T Ran S Kravtsov V Nadaf S. Vascular endothelial growth factor inhibits the development of dendritic cells and dramatically affects the differentiation of multiple hematopoietic lineages *in vivo*. Blood. (1998) 92:4150–66. 10.1182/blood.V92.11.4150.423k45_4150_4166 9834220

[59] Pollard JW. Tumour-educated macrophages promote tumour progression and metastasis. Nat Rev Cancer. (2004) 4:71–8. 10.1038/nrc1256 14708027

[60] Duluc D Delneste Y Tan F Moles MP Grimaud L Lenoir J. Tumor-associated leukemia inhibitory factor and IL-6 skew monocyte differentiation into tumor-associated macrophage-like cells. Blood. (2007) 110:4319–30. 10.1182/blood-2007-02-072587 17848619

[61] Mantovani A Sica A Sozzani S Allavena P Vecchi A Locati M. The chemokine system in diverse forms of macrophage activation and polarization. Trends Immunol. (2004) 25:677–86. 10.1016/j.it.2004.09.015 15530839

[62] Netea-Maier RT Smit JWA Netea MG. Metabolic changes in tumor cells and tumor-associated macrophages: a mutual relationship. Cancer Lett. (2018) 413:102–9. 10.1016/j.canlet.2017.10.037 29111350

[63] Mantovani A Sozzani S Locati M Allavena P Sica A. Macrophage polarization: tumor-associated macrophages as a paradigm for polarized M2 mononuclear phagocytes. Trends Immunol. (2002) 23:549–55. 10.1016/S1471-4906(02)02302-5 12401408

[64] Riabov V Gudima A Wang N Mickley A Orekhov A Kzhyshkowska J. Role of tumor associated macrophages in tumor angiogenesis and lymphangiogenesis. Front Physiol. (2014) 5:75. 10.3389/fphys.2014.00075 24634660 PMC3942647

[65] Colegio OR Chu NQ Szabo AL Chu T Rhebergen AM Jairam V. Functional polarization of tumour-associated macrophages by tumour-derived lactic acid. Nature. (2014) 513:559–63. 10.1038/nature13490 25043024 PMC4301845

[66] Munder M. Arginase: an emerging key player in the mammalian immune system. Br J Pharmacol. (2009) 158:638–51. 10.1111/j.1476-5381.2009.00291.x 19764983 PMC2765586

[67] Otsuji M Kimura Y Aoe T Okamoto Y Saito T. Oxidative stress by tumor-derived macrophages suppresses the expression of CD3 zeta chain of T-cell receptor complex and antigen-specific T-cell responses. Proc Natl Acad Sci USA. (1996) 93:13119–24. 10.1073/pnas.93.23.13119 8917554 PMC24056

[68] Kong L Zhou Y Bu H Lv T Shi Y Yang J. Deletion of interleukin-6 in monocytes/macrophages suppresses the initiation of hepatocellular carcinoma in mice. J Exp Clin Cancer Res. (2016) 35:131. 10.1186/s13046-016-0412-1 27589954 PMC5009700

[69] Qian BZ Pollard JW. Macrophage diversity enhances tumor progression and metastasis. Cell. (2010) 141:39–51. 10.1016/j.cell.2010.03.014 20371344 PMC4994190

[70] Cassetta L Fragkogianni S Sims AH Swierczak A Forrester LM Zhang H. Human tumor-associated macrophage and monocyte transcriptional landscapes reveal cancer-specific reprogramming, biomarkers, therapeutic targets. Cancer Cell. (2019) 35:588–602.e10. 10.1016/j.ccell.2019.02.009 30930117 PMC6472943

[71] Noy R Pollard JW. Tumor-associated macrophages: from mechanisms to therapy. Immunity. (2014) 41:49–61. 10.1016/j.immuni.2014.06.010 25035953 PMC4137410

[72] Martinenaite E Munir Ahmad S Hansen M Met O Westergaard MW Larsen SK. CCL22-specific T cells: modulating the immunosuppressive tumor microenvironment. Oncoimmunology. (2016) 5:e1238541. 10.1080/2162402X.2016.1238541 27999757 PMC5139648

[73] Hao NB Lu MH Fan YH Cao YL Zhang ZR Yang SM. Macrophages in tumor microenvironments and the progression of tumors. Clin Dev Immunol. (2012) 2012:948098. 10.1155/2012/948098 22778768 PMC3385963

[74] Gordon SR Maute RL Dulken BW Hutter G George BM McCracken MN. PD-1 expression by tumour-associated macrophages inhibits phagocytosis and tumour immunity. Nature. (2017) 545:495–9. 10.1038/nature22396 28514441 PMC5931375

[75] Karasuyama H Mukai K Obata K Tsujimura Y Wada T. Nonredundant roles of basophils in immunity. Annu Rev Immunol. (2011) 29:45–69. 10.1146/annurev-immunol-031210-101257 21166539

[76] Voehringer D. The role of basophils in helminth infection. Trends Parasitol. (2009) 25:551–6. 10.1016/j.pt.2009.09.004 19782643

[77] Merluzzi S Betto E Ceccaroni AA Magris R Giunta M Mion F. Mast cells, basophils and B cell connection network. Mol Immunol. (2015) 63:94–103. 10.1016/j.molimm.2014.02.016 24671125

[78] Miyake K Shiozawa N Nagao T Yoshikawa S Yamanishi Y Karasuyama H. Trogocytosis of peptide-MHC class II complexes from dendritic cells confers antigen-presenting ability on basophils. Proc Natl Acad Sci USA. (2017) 114:1111–6. 10.1073/pnas.1615973114 28096423 PMC5293035

[79] Sharma M Das M Stephen-Victor E Galeotti C Karnam A Maddur MS. Regulatory T cells induce activation rather than suppression of human basophils. Sci Immunol. (2018) 3:eaan0829. 10.1126/sciimmunol.aan0829 29802207

[80] Varricchi G Loffredo S Galdiero MR Marone G Cristinziano L Granata F. Innate effector cells in angiogenesis and lymphangiogenesis. Curr Opin Immunol. (2018) 53:152–60. 10.1016/j.coi.2018.05.002 29778674

[81] Khan ANH Emmons TR Wong JT Alqassim E Singel KL Mark J. Quantification of early-stage myeloid-derived suppressor cells in cancer requires excluding basophils. Cancer Immunol Res. (2020) 10.1158/2326-6066.CIR-19-0556. [Epub ahead of print]. 32238380 PMC7269807

[82] Stankovic B Bjorhovde HAK Skarshaug R Aamodt H Frafjord A Muller E. Immune cell composition in human non-small cell lung cancer. Front Immunol. (2018) 9:3101. 10.3389/fimmu.2018.03101 30774636 PMC6367276

[83] Afferni C Buccione C Andreone S Galdiero MR Varricchi G Marone G. The pleiotropic immunomodulatory functions of IL-33 and its implications in tumor immunity. Front Immunol. (2018) 9:2601. 10.3389/fimmu.2018.02601 30483263 PMC6242976

[84] Lambrecht BN Hammad H. The immunology of asthma. Nat Immunol. (2015) 16:45–56. 10.1038/ni.3049 25521684

[85] Mehlotra RK Hall LR Higgins AW Dreshaj IA Haxhiu MA Kazura JW. Interleukin-12 suppresses filaria-induced pulmonary eosinophilia, deposition of major basic protein and airway hyperresponsiveness. Parasite Immunol. (1998) 20:455–62. 10.1046/j.1365-3024.1998.00174.x 9797506 PMC4469192

[86] Weller PF Spencer LA. Functions of tissue-resident eosinophils. Nat Rev Immunol. (2017) 17:746–60. 10.1038/nri.2017.95 28891557 PMC5783317

[87] Yousefi S Gold JA Andina N Lee JJ Kelly AM Kozlowski E. Catapult-like release of mitochondrial DNA by eosinophils contributes to antibacterial defense. Nat Med. (2008) 14:949–53. 10.1038/nm.1855 18690244

[88] Bandeira-Melo C Bozza PT Weller PF. The cellular biology of eosinophil eicosanoid formation and function. J Allergy Clin Immunol. (2002) 109:393–400. 10.1067/mai.2002.121529 11897981

[89] Kato Y Fujisawa T Nishimori H Katsumata H Atsuta J Iguchi K. Leukotriene D4 induces production of transforming growth factor-beta1 by eosinophils. Int Arch Allergy Immunol. (2005) 137(Suppl 1):17–20. 10.1159/000085427 15947480

[90] Saito K Nagata M Kikuchi I Sakamoto Y. Leukotriene D4 and eosinophil transendothelial migration, superoxide generation, and degranulation via beta2 integrin. Ann Allergy Asthma Immunol. (2004) 93:594–600. 10.1016/S1081-1206(10)61269-0 15609771

[91] Shi HZ. Eosinophils function as antigen-presenting cells. J Leukoc Biol. (2004) 76:520–7. 10.1189/jlb.0404228 15218055

[92] Lotfi R Lotze MT. Eosinophils induce DC maturation, regulating immunity. J Leukoc Biol. (2008) 83:456–60. 10.1189/jlb.0607366 17991762

[93] Yang D Rosenberg HF Chen Q Dyer KD Kurosaka K Oppenheim JJ. Eosinophil-derived neurotoxin (EDN), an antimicrobial protein with chemotactic activities for dendritic cells. Blood. (2003) 102:3396–403. 10.1182/blood-2003-01-0151 12855582

[94] O'Donnell MC Ackerman SJ Gleich GJ Thomas LL. Activation of basophil and mast cell histamine release by eosinophil granule major basic protein. J Exp Med. (1983) 157:1981–91. 10.1084/jem.157.6.1981 6854212 PMC2187055

[95] Page SM Gleich GJ Roebuck KA Thomas LL. Stimulation of neutrophil interleukin-8 production by eosinophil granule major basic protein. Am J Respir Cell Mol Biol. (1999) 21:230–7. 10.1165/ajrcmb.21.2.3647 10423406

[96] Sakkal S Miller S Apostolopoulos V Nurgali K Eosinophils in cancer: favourable or unfavourable? Curr Med Chem. (2016) 23:650–66. 10.2174/0929867323666160119094313 26785997

[97] Simon SCS Utikal J Umansky V. Opposing roles of eosinophils in cancer. Cancer Immunol Immunother. (2019) 68:823–33. 10.1007/s00262-018-2255-4 30302498 PMC11028063

[98] Reichman H Itan M Rozenberg P Yarmolovski T Brazowski E Varol C. Activated eosinophils exert antitumorigenic activities in colorectal cancer. Cancer Immunol Res. (2019) 7:388–400. 10.1158/2326-6066.CIR-18-0494 30665890

[99] Arnold IC Artola-Boran M Tallon de Lara P Kyburz A Taube C Ottemann K. Eosinophils suppress Th1 responses and restrict bacterially induced gastrointestinal inflammation. J Exp Med. (2018) 215:2055–72. 10.1084/jem.20172049 29970473 PMC6080907

[100] Lingblom C Andersson J Andersson K Wenneras C. Regulatory eosinophils suppress T cells partly through galectin-10. J Immunol. (2017) 198:4672–81. 10.4049/jimmunol.1601005 28515279

[101] Young JD Peterson CG Venge P Cohn ZA. Mechanism of membrane damage mediated by human eosinophil cationic protein. Nature. (1986) 321:613–6. 10.1038/321613a0 2423882

[102] Patel S Fu S Mastio J Dominguez GA Purohit A Kossenkov A. Unique pattern of neutrophil migration and function during tumor progression. Nat Immunol. (2018) 19:1236–47. 10.1038/s41590-018-0229-5 30323345 PMC6195445

[103] Zehrer A Pick R Salvermoser M Boda A Miller M Stark K. A fundamental role of Myh9 for neutrophil migration in innate immunity. J Immunol. (2018) 201:1748–64. 10.4049/jimmunol.1701400 30068598

[104] Fridlender ZG Albelda SM Tumor-associated neutrophils: friend or foe? Carcinogenesis. (2012) 33:949–55. 10.1093/carcin/bgs123 22425643

[105] Uribe-Querol E Rosales C. Neutrophils in cancer: two sides of the same coin. J Immunol Res. (2015) 2015:983698. 10.1155/2015/983698 26819959 PMC4706937

[106] Fridlender ZG Sun J Kim S Kapoor V Cheng G Ling L. Polarization of tumor-associated neutrophil phenotype by TGF-beta: “N1” versus “N2” TAN. Cancer Cell. (2009) 16:183–94. 10.1016/j.ccr.2009.06.017 19732719 PMC2754404

[107] Wculek SK Malanchi I. Neutrophils support lung colonization of metastasis-initiating breast cancer cells. Nature. (2015) 528:413–7. 10.1038/nature16140 26649828 PMC4700594

[108] Zhou SL Zhou ZJ Hu ZQ Huang XW Wang Z Chen EB. Tumor-associated neutrophils recruit macrophages and T-regulatory cells to promote progression of hepatocellular carcinoma and resistance to sorafenib. Gastroenterology. (2016) 150:1646–58.e17. 10.1053/j.gastro.2016.02.040 26924089

[109] Manfroi B Moreaux J Righini C Ghiringhelli F Sturm N Huard B. Tumor-associated neutrophils correlate with poor prognosis in diffuse large B-cell lymphoma patients. Blood Cancer J. (2018) 8:66. 10.1038/s41408-018-0099-y 29977076 PMC6033870

[110] Wu L Saxena S Awaji M Singh RK Tumor-associated neutrophils in cancer: going pro. Cancers. (2019) 11:564. 10.3390/cancers11040564 31010242 PMC6520693

[111] Gabrilovich DI Nagaraj S. Myeloid-derived suppressor cells as regulators of the immune system. Nat Rev Immunol. (2009) 9:162–74. 10.1038/nri2506 19197294 PMC2828349

[112] Yu J Du W Yan F Wang Y Li H Cao S Myeloid-derived suppressor cells suppress antitumor immune responses through IDO expression and correlate with lymph node metastasis in patients with breast cancer. J Immunol. (2013) 190:3783–97. 10.4049/jimmunol.1201449 23440412

[113] Nagaraj S Gabrilovich DI. Regulation of suppressive function of myeloid-derived suppressor cells by CD4+ T cells. Semin Cancer Biol. (2012) 22:282–8. 10.1016/j.semcancer.2012.01.010 22313876 PMC3349790

[114] Mundy-Bosse BL Lesinski GB Jaime-Ramirez AC Benninger K Khan M Kuppusamy P. Myeloid-derived suppressor cell inhibition of the IFN response in tumor-bearing mice. Cancer Res. (2011) 71:5101–10. 10.1158/0008-5472.CAN-10-2670 21680779 PMC3148319

[115] Wang Y Schafer CC Hough KP Tousif S Duncan SR Kearney JF. Myeloid-derived suppressor cells impair B cell responses in lung cancer through IL-7 and STAT5. J Immunol. (2018) 201:278–95. 10.4049/jimmunol.1701069 29752311 PMC6008229

[116] Xiang X Poliakov A Liu C Liu Y Deng ZB Wang J. Induction of myeloid-derived suppressor cells by tumor exosomes. Int J Cancer. (2009) 124:2621–33. 10.1002/ijc.24249 19235923 PMC2757307

[117] Lee CR Kwak Y Yang T Han JH Park SH Ye MB. Myeloid-derived suppressor cells are controlled by regulatory T cells via TGF-beta during murine colitis. Cell Rep. (2016) 17:3219–32. 10.1016/j.celrep.2016.11.062 28009291

[118] Martin RK Saleem SJ Folgosa L Zellner HB Damle SR Nguyen GK. Mast cell histamine promotes the immunoregulatory activity of myeloid-derived suppressor cells. J Leukoc Biol. (2014) 96:151–9. 10.1189/jlb.5A1213-644R 24610880 PMC4056279

[119] Kobayashi N Hiraoka N Yamagami W Ojima H Kanai Y Kosuge T. FOXP3+ regulatory T cells affect the development and progression of hepatocarcinogenesis. Clin Cancer Res. (2007) 13:902–11. 10.1158/1078-0432.CCR-06-2363 17289884

[120] Palomares O Martin-Fontecha M Lauener R Traidl-Hoffmann C Cavkaytar O Akdis M. Regulatory T cells and immune regulation of allergic diseases: roles of IL-10 and TGF-beta. Genes Immun. (2014) 15:511–20. 10.1038/gene.2014.45 25056447

[121] Owen DL Mahmud SA Sjaastad LE Williams JB Spanier JA Simeonov DR. Thymic regulatory T cells arise via two distinct developmental programs. Nat Immunol. (2019) 20:195–205. 10.1038/s41590-018-0289-6 30643267 PMC6650268

[122] Wang R-F. CD8+ regulatory T cells, their suppressive mechanisms, and regulation in cancer. Human Immunol. (2008) 69:811–4. 10.1016/j.humimm.2008.08.276 18817828

[123] Levine AG Arvey A Jin W Rudensky AY. Continuous requirement for the TCR in regulatory T cell function. Nat Immunol. (2014) 15:1070–8. 10.1038/ni.3004 25263123 PMC4205268

[124] Thornton AM Piccirillo CA Shevach EM. Activation requirements for the induction of CD4+CD25+ T cell suppressor function. Eur J Immunol. (2004) 34:366–76. 10.1002/eji.200324455 14768041

[125] Togashi Y Shitara K Nishikawa H. Regulatory T cells in cancer immunosuppression - implications for anticancer therapy. Nat Rev Clin Oncol. (2019) 16:356–71. 10.1038/s41571-019-0175-7 30705439

[126] Jang JE Hajdu CH Liot C Miller G Dustin ML Bar-Sagi D. Crosstalk between regulatory T cells and tumor-associated dendritic cells negates anti-tumor immunity in pancreatic cancer. Cell Rep. (2017) 20:558–71. 10.1016/j.celrep.2017.06.062 28723561 PMC5649374

[127] Akkaya B Oya Y Akkaya M Al Souz J Holstein AH Kamenyeva O. Regulatory T cells mediate specific suppression by depleting peptide-MHC class II from dendritic cells. Nat Immunol. (2019) 20:218–31. 10.1038/s41590-018-0280-2 30643268 PMC6402611

[128] Lindqvist CA Christiansson LH Simonsson B Enblad G Olsson-Stromberg U Loskog AS. T regulatory cells control T-cell proliferation partly by the release of soluble CD25 in patients with B-cell malignancies. Immunology. (2010) 131:371–6. 10.1111/j.1365-2567.2010.03308.x 20518821 PMC2996557

[129] Turnis ME Sawant DV Szymczak-Workman AL Andrews LP Delgoffe GM Yano H. Interleukin-35 limits anti-tumor immunity. Immunity. (2016) 44:316–29. 10.1016/j.immuni.2016.01.013 26872697 PMC4758699

[130] Smyth MJ Teng MW Swann J Kyparissoudis K Godfrey DI Hayakawa Y. CD4+CD25+ T regulatory cells suppress NK cell-mediated immunotherapy of cancer. J Immunol. (2006) 176:1582–7. 10.4049/jimmunol.176.3.1582 16424187

[131] Xu A Liu Y Chen W Wang J Xue Y Huang F. TGF-beta-induced regulatory T cells directly suppress B cell responses through a noncytotoxic mechanism. J Immunol. (2016) 196:3631–41. 10.4049/jimmunol.1501740 27001954 PMC4868785

[132] Wilke CM Wu K Zhao E Wang G Zou W. Prognostic significance of regulatory T cells in tumor. Int J Cancer. (2010) 127:748–58. 10.1002/ijc.25464 20473951

[133] Nieman KM Romero IL Van Houten B Lengyel E. Adipose tissue and adipocytes support tumorigenesis and metastasis. Biochim Biophys Acta. (2013) 1831:1533–41. 10.1016/j.bbalip.2013.02.010 23500888 PMC3742583

[134] Bussard KM Mutkus L Stumpf K Gomez-Manzano C Marini FC Tumor-associated stromal cells as key contributors to the tumor microenvironment. Breast Cancer Res. (2016) 18:84. 10.1186/s13058-016-0740-2 27515302 PMC4982339

[135] Dirat B Bochet L Dabek M Daviaud D Dauvillier S Majed B. Cancer-associated adipocytes exhibit an activated phenotype and contribute to breast cancer invasion. Cancer Res. (2011) 71:2455–65. 10.1158/0008-5472.CAN-10-3323 21459803

[136] Fain JN. Release of interleukins and other inflammatory cytokines by human adipose tissue is enhanced in obesity and primarily due to the nonfat cells. Vitam Horm. (2006) 74:443–77. 10.1016/S0083-6729(06)74018-3 17027526

[137] Arendt LM McCready J Keller PJ Baker DD Naber SP Seewaldt V. Obesity promotes breast cancer by CCL2-mediated macrophage recruitment and angiogenesis. Cancer Res. (2013) 73:6080–93. 10.1158/0008-5472.CAN-13-0926 23959857 PMC3824388

[138] Perez VL Henault L Lichtman AH Endothelial antigen presentation: stimulation of previously activated but not naive TCR-transgenic mouse T cells. Cell Immunol. (1998) 189:31–40. 10.1006/cimm.1998.1362 9758692

[139] De Val S Black BL. Transcriptional control of endothelial cell development. Dev Cell. (2009) 16:180–95. 10.1016/j.devcel.2009.01.014 19217421 PMC2728550

[140] Dudley AC. Tumor endothelial cells. Cold Spring Harbor Perspect Med. (2012) 2:a006536. 10.1101/cshperspect.a006536 22393533 PMC3282494

[141] De Sanctis F Ugel S Facciponte J Facciabene A. The dark side of tumor-associated endothelial cells. Semin Immunol. (2018) 35:35–47. 10.1016/j.smim.2018.02.002 29490888

[142] Huang H Langenkamp E Georganaki M Loskog A Fuchs PF Dieterich LC. VEGF suppresses T-lymphocyte infiltration in the tumor microenvironment through inhibition of NF-kappaB-induced endothelial activation. FASEB J. (2015) 29:227–38. 10.1096/fj.14-250985 25361735

[143] Bergers G Song S. The role of pericytes in blood-vessel formation and maintenance. Neuro Oncol. (2005) 7:452–64. 10.1215/S1152851705000232 16212810 PMC1871727

[144] Raza A Franklin MJ Dudek AZ. Pericytes and vessel maturation during tumor angiogenesis and metastasis. Am J Hematol. (2010) 85:593–8. 10.1002/ajh.21745 20540157

[145] Harrell CR Simovic Markovic B Fellabaum C Arsenijevic A Djonov V Volarevic V. Molecular mechanisms underlying therapeutic potential of pericytes. J Biomed Sci. (2018) 25:21. 10.1186/s12929-018-0423-7 29519245 PMC5844098

[146] Bose A Barik S Banerjee S Ghosh T Mallick A Bhattacharyya Majumdar S. Tumor-derived vascular pericytes anergize Th cells. J Immunol. (2013) 191:971–81. 10.4049/jimmunol.1300280 23785117

[147] Sena IFG Paiva AE Prazeres P Azevedo PO Lousado L Bhutia SK. Glioblastoma-activated pericytes support tumor growth via immunosuppression. Cancer Med. (2018) 7:1232–9. 10.1002/cam4.1375 29479841 PMC5911609

[148] Buechler MB Turley SJ. A short field guide to fibroblast function in immunity. Semin Immunol. (2018) 35:48–58. 10.1016/j.smim.2017.11.001 29198601

[149] Van Linthout S Miteva K Tschope C. Crosstalk between fibroblasts and inflammatory cells. Cardiovasc Res. (2014) 102:258–69. 10.1093/cvr/cvu062 24728497

[150] Bu L Baba H Yoshida N Miyake K Yasuda T Uchihara T. Biological heterogeneity and versatility of cancer-associated fibroblasts in the tumor microenvironment. Oncogene. (2019) 38:4887–901. 10.1038/s41388-019-0765-y 30816343

[151] Nakamura T Matsumoto K Kiritoshi A Tano Y Nakamura T. Induction of hepatocyte growth factor in fibroblasts by tumor-derived factors affects invasive growth of tumor cells: *in vitro* analysis of tumor-stromal interactions. Cancer Res. (1997) 57:3305–13. 9242465

[152] Nakamura T Mizuno S. The discovery of hepatocyte growth factor (HGF) and its significance for cell biology, life sciences and clinical medicine. Proc Jpn Acad Ser B Phys Biol Sci. (2010) 86:588–610. 10.2183/pjab.86.588 20551596 PMC3081175

[153] Eiro N Gonzalez L Martinez-Ordonez A Fernandez-Garcia B Gonzalez LO Cid S. Cancer-associated fibroblasts affect breast cancer cell gene expression, invasion and angiogenesis. Cell Oncol. (2018) 41:369–78. 10.1007/s13402-018-0371-y 29497991 PMC12995208

[154] Donnarumma E Fiore D Nappa M Roscigno G Adamo A Iaboni M. Cancer-associated fibroblasts release exosomal microRNAs that dictate an aggressive phenotype in breast cancer. Oncotarget. (2017) 8:19592–608. 10.18632/oncotarget.14752 28121625 PMC5386708

[155] Su S Chen J Yao H Liu J Yu S Lao L. CD10(+)GPR77(+) cancer-associated fibroblasts promote cancer formation and chemoresistance by sustaining cancer stemness. Cell. (2018) 172:841–56.e16. 10.1016/j.cell.2018.01.009 29395328

[156] Benyahia Z Dussault N Cayol M Sigaud R Berenguer-Daize C Delfino C. Stromal fibroblasts present in breast carcinomas promote tumor growth and angiogenesis through adrenomedullin secretion. Oncotarget. (2017) 8:15744–62. 10.18632/oncotarget.14999 28178651 PMC5362520

[157] Takahashi H Sakakura K Kudo T Toyoda M Kaira K Oyama T. Cancer-associated fibroblasts promote an immunosuppressive microenvironment through the induction and accumulation of protumoral macrophages. Oncotarget. (2017) 8:8633–47. 10.18632/oncotarget.14374 28052009 PMC5352428

[158] Zhang A Qian Y Ye Z Chen H Xie H Zhou L. Cancer-associated fibroblasts promote M2 polarization of macrophages in pancreatic ductal adenocarcinoma. Cancer Med. (2017) 6:463–70. 10.1002/cam4.993 28097809 PMC5313646

[159] Gascard P Tlsty TD. Carcinoma-associated fibroblasts: orchestrating the composition of malignancy. Genes Dev. (2016) 30:1002–19. 10.1101/gad.279737.116 27151975 PMC4863733

[160] Costa A Kieffer Y Scholer-Dahirel A Pelon F Bourachot B Cardon M. Fibroblast heterogeneity and immunosuppressive environment in human breast cancer. Cancer Cell. (2018) 33:463–79.e10. 10.1016/j.ccell.2018.01.011 29455927

[161] Mulcahy LA Pink RC Carter DRF. Routes and mechanisms of extracellular vesicle uptake. J Extracell Vesicles. (2014) 3. 10.3402/jev.v3.24641 25143819 PMC4122821

[162] Maas SLN Breakefield XO Weaver AM. Extracellular vesicles: unique intercellular delivery vehicles. Trends Cell Biol. (2017) 27:172–88. 10.1016/j.tcb.2016.11.003 27979573 PMC5318253

[163] Comoglio PM Trusolino L Boccaccio C. Known and novel roles of the MET oncogene in cancer: a coherent approach to targeted therapy. Nat Rev Cancer. (2018) 18:341–58. 10.1038/s41568-018-0002-y 29674709

[164] Peinado H Aleckovic M Lavotshkin S Matei I Costa-Silva B Moreno-Bueno G. Melanoma exosomes educate bone marrow progenitor cells toward a pro-metastatic phenotype through MET. Nat Med. (2012) 18:883–91. 10.1038/nm.2753 22635005 PMC3645291

[165] Nakamura K Sawada K Kinose Y Yoshimura A Toda A Nakatsuka E. Exosomes promote ovarian cancer cell invasion through transfer of CD44 to peritoneal mesothelial cells. Mol Cancer Res. (2017) 15:78–92. 10.1158/1541-7786.MCR-16-0191 27758876

[166] Yue S Mu W Erb U Zoller M. The tetraspanins CD151 and Tspan8 are essential exosome components for the crosstalk between cancer initiating cells and their surrounding. Oncotarget. (2015) 6:2366–84. 10.18632/oncotarget.2958 25544774 PMC4385857

[167] Rai A Greening DW Chen M Xu R Ji H Simpson RJ. Exosomes derived from human primary and metastatic colorectal cancer cells contribute to functional heterogeneity of activated fibroblasts by reprogramming their proteome. Proteomics. (2019) 19:e1800148. 10.1002/pmic.201800148 30582284

[168] Luga V Zhang L Viloria-Petit AM Ogunjimi AA Inanlou MR Chiu E. Exosomes mediate stromal mobilization of autocrine Wnt-PCP signaling in breast cancer cell migration. Cell. (2012) 151:1542–56. 10.1016/j.cell.2012.11.024 23260141

[169] Corrado C Raimondo S Saieva L Flugy AM De Leo G Alessandro R. Exosome-mediated crosstalk between chronic myelogenous leukemia cells and human bone marrow stromal cells triggers an interleukin 8-dependent survival of leukemia cells. Cancer Lett. (2014) 348:71–6. 10.1016/j.canlet.2014.03.009 24657661

[170] Li L Tian H Chen Z Yue W Li S Li W. Inhibition of lung cancer cell proliferation mediated by human mesenchymal stem cells. Acta Biochim Biophys Sin. (2011) 43:143–8. 10.1093/abbs/gmq118 21196449

[171] Zhu W Huang L Li Y Zhang X Gu J Yan Y. Exosomes derived from human bone marrow mesenchymal stem cells promote tumor growth *in vivo*. Cancer Lett. (2012) 315:28–37. 10.1016/j.canlet.2011.10.002 22055459

[172] Haga H Yan IK Takahashi K Wood J Zubair A Patel T. Tumour cell-derived extracellular vesicles interact with mesenchymal stem cells to modulate the microenvironment and enhance cholangiocarcinoma growth. J Extracell Vesicles. (2015) 4:24900. 10.3402/jev.v4.24900 25557794 PMC4283029

[173] Nazarenko I Rana S Baumann A McAlear J Hellwig A Trendelenburg M. Cell surface tetraspanin Tspan8 contributes to molecular pathways of exosome-induced endothelial cell activation. Cancer Res. (2010) 70:1668–78. 10.1158/0008-5472.CAN-09-2470 20124479

[174] Chowdhury R Webber JP Gurney M Mason MD Tabi Z Clayton A. Cancer exosomes trigger mesenchymal stem cell differentiation into pro-angiogenic and pro-invasive myofibroblasts. Oncotarget. (2015) 6:715–31. 10.18632/oncotarget.2711 25596732 PMC4359250

[175] Ludwig N Yerneni SS Razzo BM Whiteside TL. Exosomes from HNSCC promote angiogenesis through reprogramming of endothelial cells. Mol Cancer Res. (2018) 16:1798–808. 10.1158/1541-7786.MCR-18-0358 30042174

[176] Maji S Chaudhary P Akopova I Nguyen PM Hare RJ Gryczynski I. Exosomal annexin II promotes angiogenesis and breast cancer metastasis. Mol Cancer Res. (2017) 15:93–105. 10.1158/1541-7786.MCR-16-0163 27760843 PMC5215956

[177] Al-Nedawi K Meehan B Kerbel RS Allison AC Rak J. Endothelial expression of autocrine VEGF upon the uptake of tumor-derived microvesicles containing oncogenic EGFR. Proc Natl Acad Sci USA. (2009) 106:3794–9. 10.1073/pnas.0804543106 19234131 PMC2656159

[178] Lugini L Cecchetti S Huber V Luciani F Macchia G Spadaro F. Immune surveillance properties of human NK cell-derived exosomes. J Immunol. (2012) 189:2833–42. 10.4049/jimmunol.1101988 22904309

[179] Wu C-H Li J Li L Sun J Fabbri M Wayne AS. Extracellular vesicles derived from natural killer cells use multiple cytotoxic proteins and killing mechanisms to target cancer cells. J Extracell Vesicles. (2019) 8:1588538. 10.1080/20013078.2019.1588538 30891164 PMC6419691

[180] Zhu L Kalimuthu S Gangadaran P Oh JM Lee HW Baek SH. Exosomes derived from natural killer cells exert therapeutic effect in melanoma. Theranostics. (2017) 7:2732–45. 10.7150/thno.18752 28819459 PMC5558565

[181] Fu W Lei C Liu S Cui Y Wang C Qian K. CAR exosomes derived from effector CAR-T cells have potent antitumour effects and low toxicity. Nat Commun. (2019) 10:4355. 10.1038/s41467-019-12321-3 31554797 PMC6761190

[182] Wu S-W Li L Wang Y Xiao Z. CTL-derived exosomes enhance the activation of CTLs stimulated by low-affinity peptides. Front Immunol. (2019) 10:1274. 10.3389/fimmu.2019.01274 31275303 PMC6593274

[183] Ashiru O Boutet P Fernandez-Messina L Aguera-Gonzalez S Skepper JN Vales-Gomez M. Natural killer cell cytotoxicity is suppressed by exposure to the human NKG2D ligand MICA^*^008 that is shed by tumor cells in exosomes. Cancer Res. (2010) 70:481–9. 10.1158/0008-5472.CAN-09-1688 20068167 PMC2817492

[184] Berchem G Noman MZ Bosseler M Paggetti J Baconnais S Le Cam E. Hypoxic tumor-derived microvesicles negatively regulate NK cell function by a mechanism involving TGF-β and miR23a transfer. Oncoimmunology. (2015) 5:e1062968. 10.1080/2162402X.2015.1062968 27141372 PMC4839360

[185] Wang X Shen H Zhangyuan G Huang R Zhang W He Q. 14-3-3zeta delivered by hepatocellular carcinoma-derived exosomes impaired anti-tumor function of tumor-infiltrating T lymphocytes. Cell Death Dis. (2018) 9:159. 10.1038/s41419-017-0180-7 29415983 PMC5833352

[186] Kelleher RJ Jr Balu-Iyer S Loyall J Sacca AJ Shenoy GN Peng P. Extracellular vesicles present in human ovarian tumor microenvironments induce a phosphatidylserine-dependent arrest in the T-cell signaling cascade. Cancer Immunol Res. (2015) 3:1269–78. 10.1158/2326-6066.CIR-15-0086 26112921 PMC4636911

[187] Clayton A Al-Taei S Webber J Mason MD Tabi Z. Cancer exosomes express CD39 and CD73, which suppress T cells through adenosine production. J Immunol. (2011) 187:676–83. 10.4049/jimmunol.1003884 21677139

[188] Poggio M Hu T Pai CC Chu B Belair CD Chang A. Suppression of exosomal PD-L1 induces systemic anti-tumor immunity and memory. Cell. (2019) 177:414–27.e13. 10.1016/j.cell.2019.02.016 30951669 PMC6499401

[189] Battke C Ruiss R Welsch U Wimberger P Lang S Jochum S. Tumour exosomes inhibit binding of tumour-reactive antibodies to tumour cells and reduce ADCC. Cancer Immunol Immunother. (2011) 60:639–48. 10.1007/s00262-011-0979-5 21293856 PMC11029199

[190] Zhang X Shi H Yuan X Jiang P Qian H Xu W. Tumor-derived exosomes induce N2 polarization of neutrophils to promote gastric cancer cell migration. Mol Cancer. (2018) 17:146. 10.1186/s12943-018-0898-6 30292233 PMC6174070

[191] Chalmin F Ladoire S Mignot G Vincent J Bruchard M Remy-Martin JP. Membrane-associated Hsp72 from tumor-derived exosomes mediates STAT3-dependent immunosuppressive function of mouse and human myeloid-derived suppressor cells. J Clin Invest. (2010) 120:457–71. 10.1172/JCI40483 20093776 PMC2810085

[192] Mrizak D Martin N Barjon C Jimenez-Pailhes AS Mustapha R Niki T. Effect of nasopharyngeal carcinoma-derived exosomes on human regulatory T cells. J Natl Cancer Inst. (2015) 107:363. 10.1093/jnci/dju363 25505237

[193] Szajnik M Czystowska M Szczepanski MJ Mandapathil M Whiteside TL. Tumor-derived microvesicles induce, expand and up-regulate biological activities of human regulatory T cells (Treg). PLoS ONE. (2010) 5:e11469. 10.1371/journal.pone.0011469 20661468 PMC2908536

[194] Wu L Zhang X Zhang B Shi H Yuan X Sun Y. Exosomes derived from gastric cancer cells activate NF-kappaB pathway in macrophages to promote cancer progression. Tumour Biol. (2016) 37:12169–80. 10.1007/s13277-016-5071-5 27220495

[195] Park JE Dutta B Tse SW Gupta N Tan CF Low JK. Hypoxia-induced tumor exosomes promote M2-like macrophage polarization of infiltrating myeloid cells and microRNA-mediated metabolic shift. Oncogene. (2019) 38:5158–73. 10.1038/s41388-019-0782-x 30872795

[196] Cheng L Sharples RA Scicluna BJ Hill AF. Exosomes provide a protective and enriched source of miRNA for biomarker profiling compared to intracellular and cell-free blood. J Extracell Vesicles. (2014) 3. 10.3402/jev.v3.23743 24683445 PMC3968297

[197] Schwarzenbach H Gahan PB. MicroRNA shuttle from cell-to-cell by exosomes and its impact in cancer. Noncoding RNA. (2019) 5:28. 10.3390/ncrna5010028 30901915 PMC6468647

[198] Challagundla KB Wise PM Neviani P Chava H Murtadha M Xu T. Exosome-mediated transfer of microRNAs within the tumor microenvironment and neuroblastoma resistance to chemotherapy. J Natl Cancer Inst. (2015) 107:djv135. 10.1093/jnci/djv135 25972604 PMC4651042

[199] Kosaka N Iguchi H Hagiwara K Yoshioka Y Takeshita F Ochiya T. Neutral sphingomyelinase 2 (nSMase2)-dependent exosomal transfer of angiogenic microRNAs regulate cancer cell metastasis. J Biol Chem. (2013) 288:10849–59. 10.1074/jbc.M112.446831 23439645 PMC3624465

[200] Ye SB Zhang H Cai TT Liu YN Ni JJ He J. Exosomal miR-24-3p impedes T-cell function by targeting FGF11 and serves as a potential prognostic biomarker for nasopharyngeal carcinoma. J Pathol. (2016) 240:329–40. 10.1002/path.4781 27538493

[201] Chen X Ying X Wang X Wu X Zhu Q Wang X. Exosomes derived from hypoxic epithelial ovarian cancer deliver microRNA-940 to induce macrophage M2 polarization. Oncol Rep. (2017) 38:522–8. 10.3892/or.2017.5697 28586039

[202] Wang X Luo G Zhang K Cao J Huang C Jiang T. Hypoxic tumor-derived exosomal miR-301a mediates M2 macrophage polarization via PTEN/PI3Kgamma to promote pancreatic cancer metastasis. Cancer Res. (2018) 78:4586–98. 10.1158/0008-5472.CAN-17-3841 29880482

[203] Ying X Wu Q Wu X Zhu Q Wang X Jiang L. Epithelial ovarian cancer-secreted exosomal miR-222-3p induces polarization of tumor-associated macrophages. Oncotarget.(2016) 7:43076–87. 10.18632/oncotarget.9246 27172798 PMC5190009

[204] Cai Q Zhu A Gong L. Exosomes of glioma cells deliver miR-148a to promote proliferation and metastasis of glioblastoma via targeting CADM1. Bull Cancer. (2018) 105:643–51. 10.1016/j.bulcan.2018.05.003 29921422

[205] Yang H Fu H Wang B Zhang X Mao J Li X. Exosomal miR-423-5p targets SUFU to promote cancer growth and metastasis and serves as a novel marker for gastric cancer. Mol Carcinog. (2018) 57:1223–36. 10.1002/mc.22838 29749061

[206] Yang M Chen J Su F Yu B Su F Lin L. Microvesicles secreted by macrophages shuttle invasion-potentiating microRNAs into breast cancer cells. Mol Cancer. (2011) 10:117. 10.1186/1476-4598-10-117 21939504 PMC3190352

[207] Conigliaro A Costa V Lo Dico A Saieva L Buccheri S Dieli F. CD90+ liver cancer cells modulate endothelial cell phenotype through the release of exosomes containing H19 lncRNA. Mol Cancer. (2015) 14:155. 10.1186/s12943-015-0426-x 26272696 PMC4536801

[208] Xue M Chen W Xiang A Wang R Chen H Pan J. Hypoxic exosomes facilitate bladder tumor growth and development through transferring long non-coding RNA-UCA1. Mol Cancer. (2017) 16:143. 10.1186/s12943-017-0714-8 28841829 PMC5574139

[209] Qiu JJ Lin XJ Tang XY Zheng TT Lin YY Hua KQ. Exosomal metastasisassociated lung adenocarcinoma transcript 1 promotes angiogenesis and predicts poor prognosis in epithelial ovarian cancer. Int J Biol Sci. (2018) 14:1960–73. 10.7150/ijbs.28048 30585260 PMC6299373

[210] Berrondo C Flax J Kucherov V Siebert A Osinski T Rosenberg A. Expression of the long non-coding RNA HOTAIR correlates with disease progression in bladder cancer and is contained in bladder cancer patient urinary exosomes. PLoS ONE. (2016) 11:e0147236. 10.1371/journal.pone.0147236 26800519 PMC4723257

[211] Warburg O. On the origin of cancer cells. Science. (1956) 123:309–14. 10.1126/science.123.3191.309 13298683

[212] Erra Diaz F Dantas E Geffner J. Unravelling the interplay between extracellular acidosis and immune cells. Mediators Inflamm. (2018) 2018:1218297. 10.1155/2018/1218297 30692870 PMC6332927

[213] Calcinotto A Filipazzi P Grioni M Iero M De Milito A Ricupito A. Modulation of microenvironment acidity reverses anergy in human and murine tumor-infiltrating T lymphocytes. Cancer Res. (2012) 72:2746–56. 10.1158/0008-5472.CAN-11-1272 22593198

[214] Pilon-Thomas S Kodumudi KN El-Kenawi AE Russell S Weber AM Luddy K. Neutralization of tumor acidity improves antitumor responses to immunotherapy. Cancer Res. (2016) 76:1381–90. 10.1158/0008-5472.CAN-15-1743 26719539 PMC4829106

[215] Chouaib S Noman MZ Kosmatopoulos K Curran MA. Hypoxic stress: obstacles and opportunities for innovative immunotherapy of cancer. Oncogene. (2017) 36:439–45. 10.1038/onc.2016.225 27345407 PMC5937267

[216] Li Y Patel SP Roszik J Qin Y. Hypoxia-driven immunosuppressive metabolites in the tumor microenvironment: new approaches for combinational immunotherapy. Front Immunol. (2018) 9:1591. 10.3389/fimmu.2018.01591 30061885 PMC6054965

[217] Geiger R Rieckmann JC Wolf T Basso C Feng Y Fuhrer T. L-Arginine modulates T cell metabolism and enhances survival and anti-tumor activity. Cell. (2016) 167:829–42.e13. 10.1016/j.cell.2016.09.031 27745970 PMC5075284

[218] Rodriguez PC Zea AH Culotta KS Zabaleta J Ochoa JB Ochoa AC. Regulation of T cell receptor CD3zeta chain expression by L-arginine. J Biol Chem. (2002) 277:21123–9. 10.1074/jbc.M110675200 11950832

[219] Szefel J Danielak A Kruszewski WJ. Metabolic pathways of L-arginine and therapeutic consequences in tumors. Adv Med Sci. (2018) 64:104–10. 10.1016/j.advms.2018.08.018 30605863

[220] Fletcher M Ramirez ME Sierra RA Raber P Thevenot P Al-Khami AA. l-Arginine depletion blunts antitumor T-cell responses by inducing myeloid-derived suppressor cells. Cancer Res. (2015) 75:275–83. 10.1158/0008-5472.CAN-14-1491 25406192 PMC4297565

[221] Hu Y Liu J Cui P Liu T Piao C Xu X. Synergistic effect of adoptive immunotherapy and docetaxel inhibits tumor growth in a mouse model. Cell Immunol. (2020) 348:104036. 10.1016/j.cellimm.2019.104036 31924315

[222] Steggerda SM Bennett MK Chen J Emberley E Huang T Janes JR. Inhibition of arginase by CB-1158 blocks myeloid cell-mediated immune suppression in the tumor microenvironment. J Immunother Cancer. (2017) 5:101. 10.1186/s40425-017-0308-4 29254508 PMC5735564

[223] Vigano S Alatzoglou D Irving M Menetrier-Caux C Caux C Romero P. Targeting adenosine in cancer immunotherapy to enhance T-cell function. Front Immunol. (2019) 10:925. 10.3389/fimmu.2019.00925 31244820 PMC6562565

[224] Cekic C Day YJ Sag D Linden J. Myeloid expression of adenosine A2A receptor suppresses T and NK cell responses in the solid tumor microenvironment. Cancer Res. (2014) 74:7250–9. 10.1158/0008-5472.CAN-13-3583 25377469 PMC4459782

[225] Young A Ngiow SF Gao Y Patch AM Barkauskas DS Messaoudene M. A2AR adenosine signaling suppresses natural killer cell maturation in the tumor microenvironment. Cancer Res. (2018) 78:1003–16. 10.1158/0008-5472.CAN-17-2826 29229601

[226] Isla Larrain MT Rabassa ME Lacunza E Barbera A Creton A Segal-Eiras A. IDO is highly expressed in breast cancer and breast cancer-derived circulating microvesicles and associated to aggressive types of tumors by *in silico* analysis. Tumour Biol. (2014) 35:6511–9. 10.1007/s13277-014-1859-3 24687552

[227] Liu M Wang X Wang L Ma X Gong Z Zhang S. Targeting the IDO1 pathway in cancer: from bench to bedside. J Hematol Oncol. (2018) 11:100. 10.1186/s13045-018-0644-y 30068361 PMC6090955

[228] Peng Y-P Zhang J-J Liang W-b Tu M Lu Z-P Wei J-S. Elevation of MMP-9 and IDO induced by pancreatic cancer cells mediates natural killer cell dysfunction. BMC Cancer. (2014) 14:738. 10.1186/1471-2407-14-738 25274283 PMC4287420

[229] Brandacher G Perathoner A Ladurner R Schneeberger S Obrist P Winkler C. Prognostic value of indoleamine 2,3-dioxygenase expression in colorectal cancer: effect on tumor-infiltrating T cells. Clin Cancer Res. (2006) 12:1144–51. 10.1158/1078-0432.CCR-05-1966 16489067

[230] Okamoto A Nikaido T Ochiai K Takakura S Saito M Aoki Y. Indoleamine 2,3-dioxygenase serves as a marker of poor prognosis in gene expression profiles of serous ovarian cancer cells. Clin Cancer Res. (2005) 11:6030–9. 10.1158/1078-0432.CCR-04-2671 16115948

[231] Weinlich G Murr C Richardsen L Winkler C Fuchs D. Decreased serum tryptophan concentration predicts poor prognosis in malignant melanoma patients. Dermatology. (2007) 214:8–14. 10.1159/000096906 17191041

[232] Holmgaard RB Zamarin D Li Y Gasmi B Munn DH Allison JP. Tumor-expressed IDO recruits and activates MDSCs in a Treg-dependent manner. Cell Rep. (2015) 13:412–24. 10.1016/j.celrep.2015.08.077 26411680 PMC5013825

[233] Hornyák L Dobos N Koncz G Karányi Z Páll D Szabó Z. The role of indoleamine-2,3-dioxygenase in cancer development, diagnostics, and therapy. Front Immunol. (2018) 9:151. 10.3389/fimmu.2018.00151 29445380 PMC5797779

[234] Li R Wei F Yu J Li H Ren X Hao X. IDO inhibits T-cell function through suppressing Vav1 expression and activation. Cancer Biol Ther. (2009) 8:1402–8. 10.4161/cbt.8.14.8882 19597340

[235] Munn DH Mellor AL. IDO in the tumor microenvironment: inflammation, counter-regulation, and tolerance. Trends Immunol. (2016) 37:193–207. 10.1016/j.it.2016.01.002 26839260 PMC4916957

[236] Ninomiya S Narala N Huye L Yagyu S Savoldo B Dotti G. Tumor indoleamine 2,3-dioxygenase (IDO) inhibits CD19-CAR T cells and is downregulated by lymphodepleting drugs. Blood. (2015) 125:3905–16. 10.1182/blood-2015-01-621474 25940712 PMC4473118

[237] Prendergast GC Malachowski WP DuHadaway JB Muller AJ. Discovery of IDO1 inhibitors: from bench to bedside. Cancer Res. (2017) 77:6795–811. 10.1158/0008-5472.CAN-17-2285 29247038 PMC6021761

[238] Uyttenhove C Pilotte L Theate I Stroobant V Colau D Parmentier N. Evidence for a tumoral immune resistance mechanism based on tryptophan degradation by indoleamine 2,3-dioxygenase. Nat Med. (2003) 9:1269–74. 10.1038/nm934 14502282

[239] Brown JA Dorfman DM Ma FR Sullivan EL Munoz O Wood CR. Blockade of programmed death-1 ligands on dendritic cells enhances T cell activation and cytokine production. J Immunol. (2003) 170:1257–66. 10.4049/jimmunol.170.3.1257 12538684

[240] Long GV Dummer R Hamid O Gajewski TF Caglevic C Dalle S. Epacadostat plus pembrolizumab versus placebo plus pembrolizumab in patients with unresectable or metastatic melanoma (ECHO-301/KEYNOTE-252): a phase 3, randomised, double-blind study. Lancet Oncol. (2019) 20:1083–97. 10.1016/S1470-2045(19)30274-8 31221619

[241] Vannini F Kashfi K Nath N. The dual role of iNOS in cancer. Redox Biol. (2015) 6:334–43. 10.1016/j.redox.2015.08.009 26335399 PMC4565017

[242] Burke AJ Sullivan FJ Giles FJ Glynn SA. The yin and yang of nitric oxide in cancer progression. Carcinogenesis. (2013) 34:503–12. 10.1093/carcin/bgt034 23354310

[243] Nagaraj S Gupta K Pisarev V Kinarsky L Sherman S Kang L. Altered recognition of antigen is a mechanism of CD8+ T cell tolerance in cancer. Nat Med. (2007) 13:828–35. 10.1038/nm1609 17603493 PMC2135607

[244] Molon B Ugel S Del Pozzo F Soldani C Zilio S Avella D. Chemokine nitration prevents intratumoral infiltration of antigen-specific T cells. J Exp Med. (2011) 208:1949–62. 10.1084/jem.20101956 21930770 PMC3182051

[245] Gao Y Zhou S Xu Y Sheng S Qian SY Huo X. Nitric oxide synthase inhibitors 1400W and L-NIO inhibit angiogenesis pathway of colorectal cancer. Nitric Oxide. (2019) 83:33–9. 10.1016/j.niox.2018.12.008 30590117 PMC6677402

[246] Saluja M Gilling P. Intravesical bacillus Calmette-Guerin instillation in non-muscle-invasive bladder cancer: a review. Int J Urol. (2018) 25:18–24. 10.1111/iju.13410 28741703

[247] Rosenberg SA Yang JC Sherry RM Kammula US Hughes MS Phan GQ. Durable complete responses in heavily pretreated patients with metastatic melanoma using T-cell transfer immunotherapy. Clin Cancer Res. (2011) 17:4550–7. 10.1158/1078-0432.CCR-11-0116 21498393 PMC3131487

[248] Kalos M Levine BL Porter DL Katz S Grupp SA Bagg A. T cells with chimeric antigen receptors have potent antitumor effects and can establish memory in patients with advanced leukemia. Sci Transl Med. (2011) 3:95ra73. 10.1126/scitranslmed.3002842 21832238 PMC3393096

[249] Zhao L Cao YJ. Engineered T cell therapy for cancer in the clinic. Front Immunol. (2019) 10:2250. 10.3389/fimmu.2019.02250 31681259 PMC6798078

[250] Ribas A Wolchok JD. Cancer immunotherapy using checkpoint blockade. Science. (2018) 359:1350–5. 10.1126/science.aar4060 29567705 PMC7391259

[251] Borcherding N Kolb R Gullicksrud J Vikas P Zhu Y Zhang W. Keeping tumors in check: a mechanistic review of clinical response and resistance to immune checkpoint blockade in cancer. J Mol Biol. (2018) 430:2014–29. 10.1016/j.jmb.2018.05.030 29800567 PMC6071324

[252] Kalbasi A Ribas A. Tumour-intrinsic resistance to immune checkpoint blockade. Nat Rev Immunol. (2019) 20:25–39. 10.1038/s41577-019-0218-4 31570880 PMC8499690

[253] Le DT Uram JN Wang H Bartlett BR Kemberling H Eyring AD. PD-1 blockade in tumors with mismatch-repair deficiency. N Engl J Med. (2015) 372:2509–20. 10.1056/NEJMoa1500596 26028255 PMC4481136

[254] Rodig SJ Gusenleitner D Jackson DG Gjini E Giobbie-Hurder A Jin C. MHC proteins confer differential sensitivity to CTLA-4 and PD-1 blockade in untreated metastatic melanoma. Sci Transl Med. (2018) 10:eaar3342. 10.1126/scitranslmed.aar3342 30021886

[255] Galluzzi L Vacchelli E Bravo-San Pedro JM Buque A Senovilla L Baracco EE. Classification of current anticancer immunotherapies. Oncotarget. (2014) 5:12472–508. 10.18632/oncotarget.2998 25537519 PMC4350348

[256] Argyle D Kitamura T. Targeting macrophage-recruiting chemokines as a novel therapeutic strategy to prevent the progression of solid tumors. Front Immunol. (2018) 9:2629. 10.3389/fimmu.2018.02629 30483271 PMC6243037

[257] Blom K Rubin J Berglund M Jarvius M Lenhammar L Parrow V. Mebendazole-induced M1 polarisation of THP-1 macrophages may involve DYRK1B inhibition. BMC Res Notes. (2019) 12:234. 10.1186/s13104-019-4273-5 31010428 PMC6477744

[258] Christiansson L Soderlund S Mangsbo S Hjorth-Hansen H Hoglund M Markevarn B. The tyrosine kinase inhibitors imatinib and dasatinib reduce myeloid suppressor cells and release effector lymphocyte responses. Mol Cancer Ther. (2015) 14:1181–91. 10.1158/1535-7163.MCT-14-0849 25761894

[259] Eriksson E Wenthe J Irenaeus S Loskog A Ullenhag G. Gemcitabine reduces MDSCs, tregs and TGFbeta-1 while restoring the teff/treg ratio in patients with pancreatic cancer. J Transl Med. (2016) 14:282. 10.1186/s12967-016-1037-z 27687804 PMC5041438

[260] Zahoor H Mir MC Barata PC Stephenson AJ Campbell SC Fergany A. Phase II trial of continuous treatment with sunitinib in patients with high-risk (BCG-refractory) non-muscle invasive bladder cancer. Invest New Drugs. (2019) 37:1231–8. 10.1007/s10637-018-00716-w 31231785

[261] Georganaki M van Hooren L Dimberg A. Vascular targeting to increase the efficiency of immune checkpoint blockade in cancer. Front Immunol. (2018) 9:3081. 10.3389/fimmu.2018.03081 30627131 PMC6309238

[262] Subudhi SK Vence L Zhao H Blando J Yadav SS Xiong Q. Neoantigen responses, immune correlates, and favorable outcomes after ipilimumab treatment of patients with prostate cancer. Sci Transl Med. (2020) 12:eaaz3577. 10.1126/scitranslmed.aaz3577 32238575

[263] Fumet JD Richard C Ledys F Klopfenstein Q Joubert P Routy B. Prognostic and predictive role of CD8 and PD-L1 determination in lung tumor tissue of patients under anti-PD-1 therapy. Br J Cancer. (2018) 119:950–60. 10.1038/s41416-018-0220-9 30318514 PMC6203820

[264] Garon EB Rizvi NA Hui R Leighl N Balmanoukian AS Eder JP. Pembrolizumab for the treatment of non-small-cell lung cancer. N Engl J Med. (2015) 372:2018–28. 10.1056/NEJMoa1501824 25891174

[265] Rosenberg JE Hoffman-Censits J Powles T van der Heijden MS Balar AV Necchi A. Atezolizumab in patients with locally advanced and metastatic urothelial carcinoma who have progressed following treatment with platinum-based chemotherapy: a single-arm, multicentre, phase 2 trial. Lancet. (2016) 387:1909–20. 10.1016/S0140-6736(16)00561-4 26952546 PMC5480242

[266] Schiza A Wenthe J Mangsbo S Eriksson E Nilsson A Totterman TH. Adenovirus-mediated CD40L gene transfer increases Teffector/Tregulatory cell ratio and upregulates death receptors in metastatic melanoma patients. J Transl Med. (2017) 15:79. 10.1186/s12967-017-1182-z 28427434 PMC5399418

[267] Tumeh PC Harview CL Yearley JH Shintaku IP Taylor EJ Robert L. PD-1 blockade induces responses by inhibiting adaptive immune resistance. Nature. (2014) 515:568–71. 10.1038/nature13954 25428505 PMC4246418

[268] Topalian SL Sznol M McDermott DF Kluger HM Carvajal RD Sharfman WH. Survival, durable tumor remission, and long-term safety in patients with advanced melanoma receiving nivolumab. J Clin Oncol. (2014) 32:1020–30. 10.1200/JCO.2013.53.0105 24590637 PMC4811023

[269] Nonomura Y Otsuka A Nakashima C Seidel JA Kitoh A. Peripheral blood Th9 cells are a possible pharmacodynamic biomarker of nivolumab treatment efficacy in metastatic melanoma patients. Oncoimmunology. (2016) 5:e1248327. 10.1080/2162402X.2016.1248327 28123885 PMC5215264

[270] Ribas A Dummer R Puzanov I VanderWalde A Andtbacka RHI Michielin O. Oncolytic virotherapy promotes intratumoral T cell infiltration and improves anti-PD-1 immunotherapy. Cell. (2017) 170:1109–19.e10. 10.1016/j.cell.2017.08.027 28886381 PMC8034392

[271] Tobin RP Jordan KR Robinson WA Davis D Borges VF Gonzalez R. Targeting myeloid-derived suppressor cells using all-trans retinoic acid in melanoma patients treated with Ipilimumab. Int Immunopharmacol. (2018) 63:282–91. 10.1016/j.intimp.2018.08.007 30121453 PMC6134177

[272] de Coana YP Wolodarski M Poschke I Yoshimoto Y Yang Y Nystrom M. Ipilimumab treatment decreases monocytic MDSCs and increases CD8 effector memory T cells in long-term survivors with advanced melanoma. Oncotarget. (2017) 8:21539–53. 10.18632/oncotarget.15368 28423487 PMC5400604

[273] Malmstrom PU Loskog AS Lindqvist CA Mangsbo SM Fransson M Wanders A. AdCD40L immunogene therapy for bladder carcinoma–the first phase I/IIa trial. Clin Cancer Res. (2010) 16:3279–87. 10.1158/1078-0432.CCR-10-0385 20448220

[274] Donskov F Bennedsgaard KM Hokland M Marcussen N Fisker R Madsen HH. Leukocyte orchestration in blood and tumour tissue following interleukin-2 based immunotherapy in metastatic renal cell carcinoma. Cancer Immunol Immunother. (2004) 53:729–39. 10.1007/s00262-004-0525-9 15088127 PMC11032892

[275] Jensen HK Donskov F Nordsmark M Marcussen N von der Maase H. Increased intratumoral FOXP3-positive regulatory immune cells during interleukin-2 treatment in metastatic renal cell carcinoma. Clin Cancer Res. (2009) 15:1052–8. 10.1158/1078-0432.CCR-08-1296 19188179

[276] Qin Z Chen J Zeng J Niu L Xie S Wang X. Effect of NK cell immunotherapy on immune function in patients with hepatic carcinoma: a preliminary clinical study. Cancer Biol Ther. (2017) 18:323–30. 10.1080/15384047.2017.1310346 28353401 PMC5499755

[277] Xie S Wu Z Niu L Chen J Ma Y Zhang M. Preparation of highly activated natural killer cells for advanced lung cancer therapy. Onco Targets Ther. (2019) 12:5077–86. 10.2147/OTT.S201924 31308687 PMC6616273

[278] Tada Y Togashi Y Kotani D Kuwata T Sato E Kawazoe A. Targeting VEGFR2 with Ramucirumab strongly impacts effector/ activated regulatory T cells and CD8(+) T cells in the tumor microenvironment. J Immunother Cancer. (2018) 6:106. 10.1186/s40425-018-0403-1 30314524 PMC6186121

[279] Terme M Pernot S Marcheteau E Sandoval F Benhamouda N Colussi O. VEGFA-VEGFR pathway blockade inhibits tumor-induced regulatory T-cell proliferation in colorectal cancer. Cancer Res. (2013) 73:539–49. 10.1158/0008-5472.CAN-12-2325 23108136

[280] Enblad G Karlsson H Gammelgard G Wenthe J Lovgren T Amini RM. A Phase I/IIa trial using CD19-targeted third-generation CAR T cells for lymphoma and leukemia. Clin Cancer Res. (2018) 24:6185–94. 10.1158/1078-0432.CCR-18-0426 30097433

[281] Quillien V Carpentier AF Gey A Avril T Tartour E Sejalon F. Absolute numbers of regulatory T cells and neutrophils in corticosteroid-free patients are predictive for response to bevacizumab in recurrent glioblastoma patients. Cancer Immunol Immunother. (2019) 68:871–82. 10.1007/s00262-019-02317-9 30830269 PMC6529384

[282] Weed DT Zilio S Reis IM Sargi Z Abouyared M Gomez-Fernandez CR. The reversal of immune exclusion mediated by tadalafil and an anti-tumor vaccine also induces PDL1 upregulation in recurrent head and neck squamous cell carcinoma: interim analysis of a phase I clinical trial. Front Immunol. (2019) 10:1206. 10.3389/fimmu.2019.01206 31214178 PMC6554471

